# Pyranone Derivatives With Antitumor Activities, From the Endophytic Fungus *Phoma* sp. YN02-P-3

**DOI:** 10.3389/fchem.2022.950726

**Published:** 2022-07-07

**Authors:** Chong Yu, Yin Nian, Huanhua Chen, Shuwen Liang, Mengyang Sun, Yuehu Pei, Haifeng Wang

**Affiliations:** ^1^ School of Traditional Chinese Materia Medica, Shenyang Pharmaceutical University, Shenyang, China; ^2^ State Key Laboratory of Phytochemistry and Plant Resources in West China, Kunming Institute of Botany, Chinese Academy of Sciences, Kunming, China; ^3^ Department of Medicinal Chemistry and Natural Medicine Chemistry, College of Pharmacy, Harbin Medical University, HarBin, China

**Keywords:** secondary metabolites, plant endophytic fungus, *Phoma* sp., pyranone derivatives, HL-60 inhibition

## Abstract

Two new pyranone derivatives phomapyrone A (2) and phomapyrone B (3), one new coumarin 11*S,* 13*R*-(+)-phomacumarin A (1), three known pyranones (4–6), together with three known amide alkaloids fuscoatramides A-C (7–9), as well as 9*S,* 11*R*-(+)-ascosalitoxin (10) were isolated from the endophytic fungus *Phoma* sp. YN02-P-3, which was isolated from the healthy leaf tissue of a Paulownia tree in Yunnan Province, China. Their structures were elucidated using extensive NMR spectroscopic and HRESIMS data and by comparing the information with literature data. In addition, all compounds were tested for their cytotoxicity activity against human tumor cell lines, and the results showed that new compounds 1-3 showed moderate inhibitory activity against the HL-60 cell line with IC_50_ values of 31.02, 34.62, and 27.90 *μ*M, respectively.

## 1 Introduction

With the increasing number of human health problems and diseases, the demand for secondary metabolites in the international market is also increasing, which poses a serious threat to many plant species (Gupta et al., 2019; Zhu et al., 2011). In recent years, endophytic fungi from plants have attracted much attention because of their indispensable diversity, unique distribution, and unique metabolic pathway (Yadav et al., 2022). Endophytic fungi, as a potential alternative source of bioactive plant metabolites, have become a promising substitute for plant secondary metabolites (Torres-mendoza et al., 2020). Furthermore, studies have shown that secondary metabolites of endophytic fungi are likely to be developed into drugs. Many promising lead compounds have been found from them, which have proved to be a treasure trove of promising new compound sources (Zheng et al., 2021). The dominating types of secondary metabolites obtained from endophytic fungi included alkaloids, peptides, terpenoids, polyketones, and steroids (Ortega et al., 2021; Singh et al., 2011; Tan et al., 2021). Moreover, some metabolites in endophytic fungi show highly selective and new mechanisms of action (Zhu et al., 2011), exhibiting antibacterial and antifungal activities (Aq et al., 2020), antiviral and antigenic animal activities (Manganyi and Ateba, 2020; Cadamuro et al., 2021), anti-inflammatory and antioxidant activities (Cruz et al., 2020), cytotoxic activity and anticancer properties (Tyagi et al., 2021), insecticidal activity (Jia et al., 2020). In addition, endophytic fungi are also promising biological herbicide and plant growth promoters (Poveda et al., 2021; Macias-Rubalcava and Garrido-Santos, 2022). In conclusion, the study of endophytic fungi and their secondary metabolites can not only protect plant resources and produce secondary metabolites sustainably, which is of great significance to environmental protection but also bring substantial benefits to the development of new drugs against human diseases (Carvalho et al., 2020).

**FIGURE 1:**
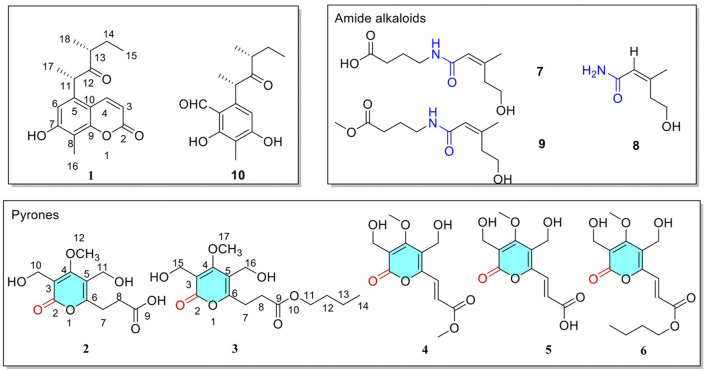
Structure of the identified compounds 1–10.

In our study, a fungus was isolated from the healthy leaf tissue of a Paulownia tree in Yunnan Province, China. The fungus was identified as *Phoma* sp. by morphological and molecular biological ITS sequences (The ITS sequence was in the supplementary materials). The structure of compounds obtained from endophytic fungus YN02-P-3 was shown in Figure 1. Three new compounds were isolated and identified via adopting two types of solid culture medium of rice and corn for fermentation. Among them, compound 1 was isolated from ethyl acetate extract of corn solid medium, and compounds 2 and 3 were separated from the *n*-butanol extract of rice medium. The new compounds carried on the structure identification. The absolute configuration of compound 1 was determined by Density Functional Theory (DFT). The cytotoxic activities of these new compounds were evaluated by MTT assay (Pimjuk et al., 2020; Noor et al., 2020), and the results showed that the three new compounds had moderate inhibitory activity against human acute promyeloid leukemia cell line HL-60, but had no significant inhibitory effect on PC-3 and HT-29 cells. The isolation, structural elucidation, and cytotoxic activity of these compounds were described herein.

## 2 Experimental

### 2.1 General Experimental Procedures

UV spectra were measured on a Shimadzu UV-2201 (Shimadzu, Tokyo, Japan). IR spectra were measured on a Bruker IFS-55 (Bruker, Karlsruhe, Germany). Bruker Micro TOF-Q mass spectrometer (Waters, MA, United States). Optical rotations were measured on Perkin-elmer 241 MC polarimeter (Perkinelmer, United States). NMR spectra were performed on Bruker ARX-600 or ARX-400 spectrometer with TMS as an internal standard (Karlsruhe, Germany). HPLC separation was carried out on a semi-preparative YMC-pack ODS-A column (250 × 10 mm, YMC-pack, Kyoto, Japan) equipped with Shimadzu LC-8A detector and Shimadzu SPD-10A UV series pumping system (Kyoto, Japan). Deuterium reagent for Cambridge Isotope Laboratories, LNC. (CIL, United States). Silica gel GF254 (200–300 mesh; Qingdao Yuwang Factory, Qingdao, China). Sephadex LH-20 (18–110 lm; Pharmacia, Piscataway, NJ, United States). ODS (50 lm; YMC Co. Kyoto, Japan).

### 2.2 Fungal Material and Fermentation

Plant samples of the fungus were collected in Daweishan, Yunnan province in June 2013, and the strain was stored in the School of Traditional Chinese Medicine, Shenyang Pharmaceutical University.

YN02-P-3 strain was purified and cultured on a PDA medium (potato 20%, glucose 2%, and agar 2%) for 3–4 days to form a round flat white colony with neat edges and orange-red on the back (Supplementary Figure S36). The purified fungus was inoculated with a high-temperature (121°C) sterilized bamboo stick into a conical flask (250 ml) containing the liquid medium of fungus No. 4, with a cotton plug, and cultured in a shaker at 25°C for 3 days at 180 r. The seed liquid of the strain was inoculated into corn and rice medium (200 vials, about 5 ml per vial). The fermentation was left at 25°C for 30 days.

### 2.3 Separation and Purification of Secondary Metabolites

The corn medium was mashed and extracted three times with ethyl acetate to obtain a crude extract of 70 g. The rice medium was pounded and extracted with *n*-butanol three times to obtain a crude extract of 47 g. The EtOAc layer was separated over a column of silica gel using PE-Acetone (100:0–0:100) to give 7 subfractions (F1 to F7). F3 (5.5 g) was subjected to ODS (MeOH: H_2_O = 0:100–100:0) to get F3-2 (2.1 g) and then F3-2 applied to pre-HPLC with MeOH/H_2_O (60/40, v/v, flow rate 2.5 ml/min) as eluent to obtain compound 1 (9.1 mg, *t*_R_ = 40 min). The *n*-butanol layer was separated over a column of silica gel using PE-Acetone (100:0–0:100) to give 7 subfractions (F1 to F7). F6 (10.5 g) was subjected to ODS (MeOH: H_2_O = 0:100–100:0) to get F6-4 (4.1 g) and then F6-4 applied to pre-HPLC with MeOH/H_2_O (40/60, v/v, flow rate 2.5 ml/min) as eluent to obtain compound 2 (8.2 mg, *t*_R_ = 56 min) and compound 3 (25.6 mg, *t*_R_ = 30 min).

#### 2.3.1 11S, 13R-(+)-Phomacumarin A (1)

Yellow oil (MeOH); UV: 329 nm, 262 nm; IR (KBr): 3364, 2970, 2932, 2876, 1703, 1595, 1575, 1493, 1394, 1298, 1120, 1004, 828, 493 cm^−1^; [*α*]^20^_D_+84.17 (c 0.1, MeOH); ^1^H-NMR (400 MHz, DMSO-*d*_6_) and ^13^C-NMR (100 MHz, DMSO-*d*_6_) data are shown in Table 1; HR-ESI-MS; *m/z* 289.1439 [M + H]^+^, (calcd for C_17_H_20_O_4_, 289.1434).

**TABLE 1:** ^1^H NMR and ^13^C NMR spectral data in DMSO-*d*_6_ for compound 1.x

Position	*δ*_H_ (*J* in Hz)	*δ*_C_
2		160.2
3	6.27 (1H, d, *J* = 9.8 Hz)	111.1
4	8.34 (1H, d, *J* = 9.8 Hz)	141.1
5		136.9
6	6.61 (1H, s)	110.9
7		159.0
8		109.8
9		154.3
10		109.5
11	4.58 (1H, q, *J* = 6.7 Hz)	44.6
12	2.35 (1H, m)	212.6
13	1.45 (1H, m)	45.7
14	1.15 (1H, m)	24.9
15	0.53 (3H, t, *J* = 7.3 Hz)	11.4
16	2.12 (3H, s)	7.8
17	1.25 (3H, d, *J* = 6.7 Hz)	18.1
18	0.99 (3H, d, *J* = 7.0 Hz)	17.0
7-OH	10.49(1H, s)	

#### 2.3.2 Phomapyrone A (2)

Yellow oil (MeOH); UV: 295 nm; IR (KBr): 3423, 2985, 2831, 1609, 1492, 1441, 1399, 1367, 1265, 1173, 1008, 799, 775, 615 cm^−1^; ^1^H-NMR (400 MHz, DMSO-*d*_6_) and ^13^C-NMR (100 MHz, DMSO-*d*_6_) data are shown in Table 2; HR-ESI-MS; *m/z* 258.0665 [M + Na]^+^, (calcd for C_11_H_14_O_7_, 258.0632).

**TABLE 2:** ^1^H NMR and ^13^C NMR spectral data in DMSO-*d*_6_ for compounds 2–3.

Position	Compound 2		Compound 3	
	*δ*_C_	*δ*_H_ (*J* in Hz)	*δ*_C_	*δ*_H_ (*J* in Hz)
2	163.9		163.8	
3	109.8		109.9	
4	168.9		168.9	
5	113.9		114.1	
6	162.0		161.5	
7	25.7	2.84 (2H, t, 7.4)	25.6	2.88 (2H, t, 7.4)
8	31.2	2.55 (2H, t, 7.4)	31.1	2.63 (2H, t, 7.4)
9	173.1		171.6	
10	53.3	4.32 (2H, s)		
11	53.2	4.26 (2H, s)	63.8	4.02 (2H, t, 6.5)
12	62.0	4.06 (3H, s)	30.1	1.53 (2H, m)
13			18.6	1.31 (2H, m)
14			13.5	0.88 (3H, t, 7.3)
15			53.2	4.32 (2H, d, 4.0)
16			53.3	4.25 (2H, d, 4.0)
17			62.0	4.06 (3H, s)
10-OH		4.96 (1H, s)		
15,16-OH				4.96 (2H, m)

#### 2.3.3 Phomapyrone B (3)

Yellow oil (MeOH); UV: 254 nm; IR (KBr): 3424, 2984, 2831, 1607, 1492, 1440, 1398, 1366, 1262, 1174, 1009, 800, 775 cm^−1^; ^1^H-NMR (400 MHz, DMSO-*d*_6_) and ^13^C-NMR (100 MHz, DMSO-*d*_6_) data are shown in Table 2; HR-ESI-MS; *m/z* 314.1269 [M + Na]^+^, (calcd for C_15_H_22_O_7_, 314.1258).

### 2.4 OR Calculations

Conformational analyses were carried out *via* random searching in the Sybyl-X 2.0 using the MMFF94S force field with an energy cut-off of 2.5 kcal/mol. Subsequently, All these conformers were initially optimized using DFT at the B3LYP-D3(BJ)/6–31G* level in PCM MeOH by the GAUSSIAN 09 program. The free energy values were obtained from the vibrational frequency calculations as the sum of electronic and thermal free energies. The Gibbs free energy equation (ΔG = -*RT* ln K) was used to obtain the Boltzmann-weighted conformer population. The OR calculations were carried out on Gaussian 09 using the DFT method at the CAM-B3LYP/6–311++G(2d,p) level in PCM (MeOH) at 589.3 nm.

### 2.5 Cytotoxic Activity

Using 5-fluorouridine as a positive control, an MTT assay was used to detect the cytotoxic activity of all compounds against HL-60. MTT was weighed, dissolved in PBS, and prepared into 2 mg/ml solution, stirred in dark for 30 min, filtered and sterilized with 0.22 *μ*M membrane, separated, and stored at −20°C. The cells in the logarithmic growth phase were inoculated in a 96-well culture plate at the density of (2–3)10^4^ cells/mL, 100 *μ*L in each well, and adhered to the wall for 24 h. After that, the cells were diluted to 100 *μ*L of different concentrations of the compound to be measured, and the culture was continued at 37°C for 96 h. Then add 50 *μ*L MTT solution to each well and incubate at 37°C for 4 h. Discard the supernatant and add 200 *μ*L DMSO to each well. After shaking at room temperature for 10 min, the absorbance value of each well was measured at 570 nm by a microplate analyzer. Then determine the IC_50_ value.

## 3 Results and Discussion

### 3.1 Structure Elucidation of Secondary Metabolites

Compound 1 was originally obtained as a yellow oil (MeOH), and its molecular formula was determined as C_17_H_20_O_4_ on the basis of HRESI-TOF-MS data (*m/z* 289.1439 [M + H]^+^), implying eight degrees of unsaturation. The ^1^H-NMR (400 MHz, DMSO-*d*_6_) spectrum (Table 1), showed one phenol hydroxyl hydrogen signal *δ*_H_ 10.49 (1H, s, 7-OH), two olefinic signals *δ*_H_ 8.34 (1H, d, *J* = 9.8 Hz, H-4), 6.27 (1H, d, *J* = 9.8 Hz, H-3), one hydrogen signal on the benzene ring *δ*_H_ 6.61 (1H, s, H-6), two methylidyne signals *δ*_H_ 4.58 (1H, q, *J* = 6.7 Hz, H-11), 2.35 (1H, m, H-13), two methylene signals *δ*_H_ 1.45 (1H, m, H-14_a_), 1.15 (1H, m, H-14_b_), four methine signals *δ*_H_ 2.12 (3H, s, H-16), 0.53 (3H, t, *J* = 7.3 Hz, H-15), 1.25 (3H, d, *J* = 6.7 Hz, H-17), 0.99 (3H, d, *J* = 7.0 Hz, H-18). The ^13^C-NMR (100 MHz, DMSO-*d*_6_) with the aid of the HSQC spectrum showed signals of carbons including one ketone carbonyl carbon *δ*_C_ 212.6 (C-12), three olefinic signals *δ*_C_ 160.2 (C-2), 141.1 (C-4), 111.1 (C-3), six aromatic carbons *δ*_C_ 159.0 (C-7), 154.3 (C-9), 136.9 (C-5), 110.9 (C-6), 109.8 (C-8), 109.5 (C-10), seven aliphatic signals *δ*_C_ 45.7 (C-13), 44.6 (C-11), 24.9 (C-14), 18.1 (C-17), 17.0 (C-18), 11.4 (C-15), 7.8 (C-16). In addition, three olefinic carbon signals suggested the presence of an oxygen atom was linked to one of the carbon atoms, so it was speculated that *α-β* unsaturated ketone fragment carbon signals. What’s more, six aromatic carbon signals indicated the presence of a benzene ring with two oxygen atoms.

**FIGURE 2:**
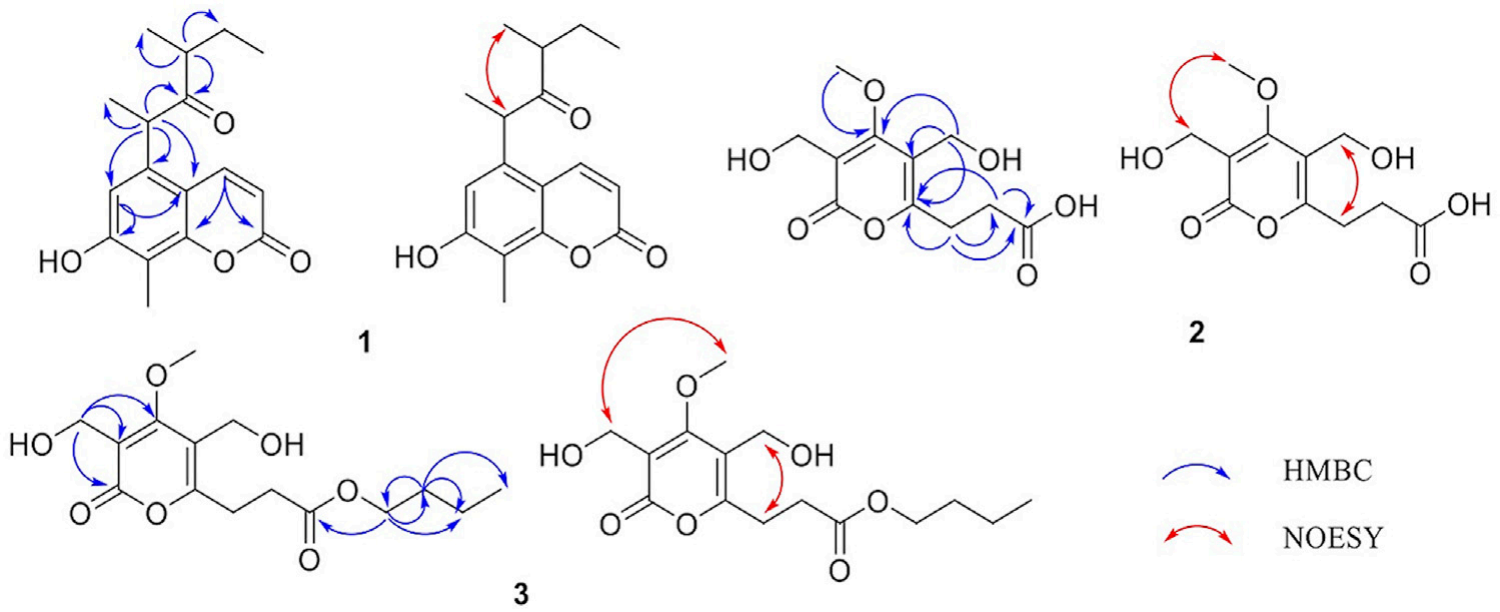
Key HMBC (blue) and NOESY (red) correlations of 1–3.

The HMBC spectrum (Figure 2) showed correlations of (H-6 with C-7/C-10/C-11 and H-4 with C-2/C-9 and H-16 with C-7/C-8/C-9), which suggested the presence of coumarin parent nucleus. The HMBC spectrum showed correlations of (H-11 with C-5/C-6/C-10/C-12/C-17 and H-13 with C-12/C-14/C-18 and H-15 with C-13/C-14), which indicated the presence of 1, 3-dimethylpentanone, and 1, 3-dimethylpentanone connected to coumarin parent nucleus via C-5 and C-11. Consequently, the plane molecular structure of the compound was determined.

**FIGURE 3:**
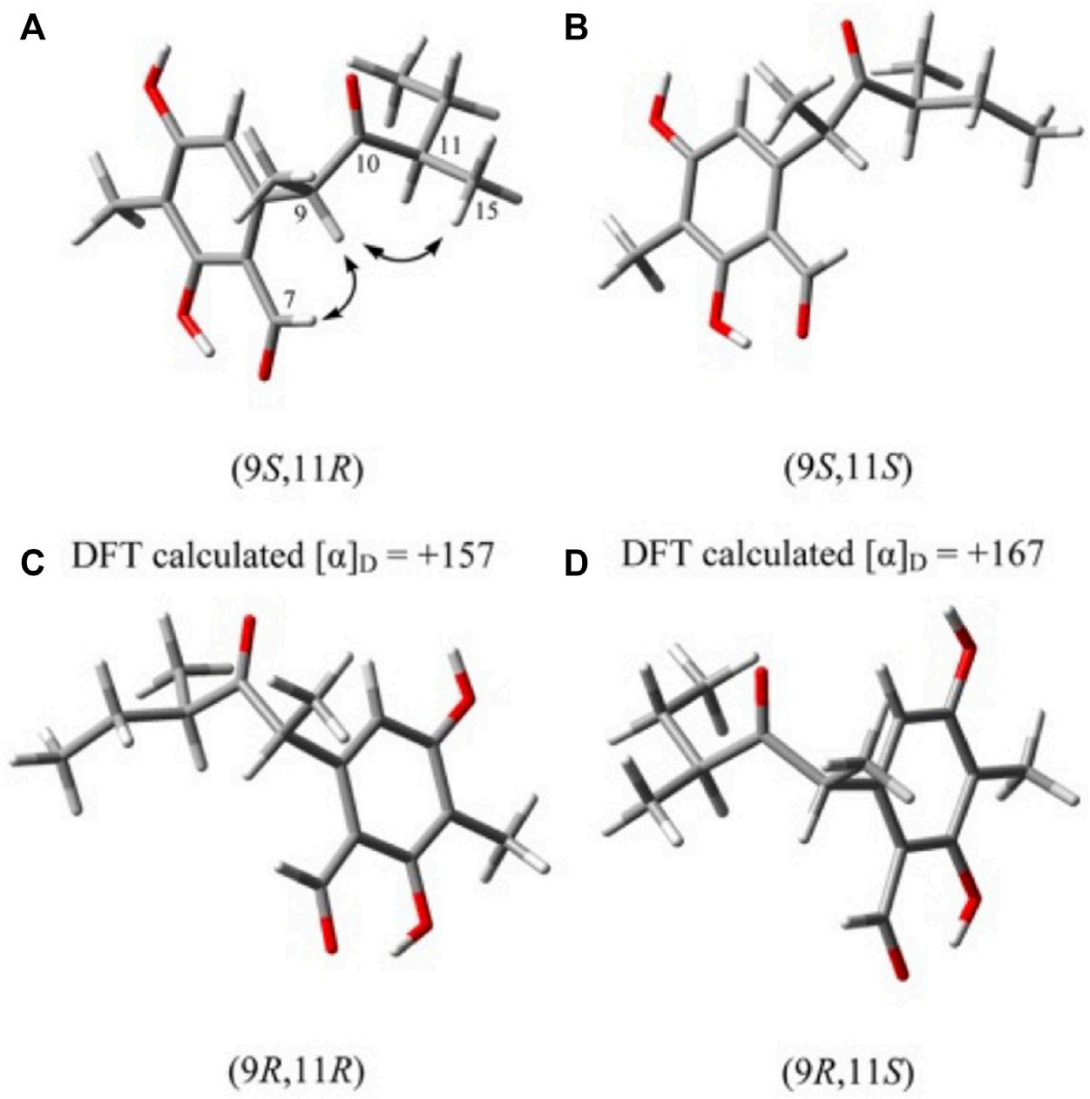
Global minimum energy structures of the four possible diastereoisomers of compound 1.

The absolute configuration of compound 1 was determined by comparing it with the known literature Liangsakul J. et al., 2012. Compound 9*S,* 11*R-(+)-ascosalitoxin* had a similar structure to compound 1. There were four potential conformations after the conformation optimization of DFT, in order to solve the conformation problem, DFT was used for OR calculations. The DFT results (Figure 3) were displayed that in the case of a (9*S*, 11*R*), [*α*]_D_ = +157, and in the case of b (9*S*,11*S*), [*α*]_D_ = +167, and in the case of c (9*R*,11*R*), [*α*]_D_ = −167, and in the case of d (9*R*,11*S*), [*α*]_D_ = −157. Both cases c and d could be ruled out by optical activity. In the lowest energy state of a, the space distance between H-9 and 15-CH_3_ was closed (mean distance of 2.4 Å), while in the case of b, the mean distance between H-9 and 15-CH_3_ was 4.7 Å. There was an obvious correlation between H-9 and 15-CH_3_ in the NOESY spectrum. Therefore, the absolute configuration of compound 1 was 9*S,* 11*R*-(+)-ascosalitoxin. Compound 1 has similar optical activity, and *δ*_H_ 4.58 (1H, q, *J* = 6.7 Hz, H-11) was correlated with *δ*_H_ 0.99 (3H, d, *J* = 7.0 Hz, H-18) in the NOESY spectrum, so it was determined to be 11*S*, 13*R*. Thus, the structure 11*S,* 13*R*-(+)-Phomacumarin A was determined, and the systematic name was *7-hydroxy-8-methyl-5-[(2S, 4R)-4-methyl-3-oxohexan-2-yl]-2H-chromen-2-one*.

Compound 2 was deduced as a yellow oil (MeOH) and was assigned a molecular formula of C_11_H_14_O_7_, which requires 5 degrees of unsaturation based on HRESIMS (*m/z* 258.0665 [M + Na]^+^). The ^1^H NMR spectrum (400 MHz, DMSO-*d*_*6*_) with the aid of the HSQC spectrum showed signals of hydrogen including one active hydroxyl signal *δ*_H_ 4.96 (1H, s, 10-OH), one methoxyl hydrogen signal [*δ*_H_ 4.06 (3H, s, -OCH_3_) was correlated with *δ*_C_ 62.0 (C-12)], two oxymethylene hydrogen signals [*δ*_H_ 4.26 (2H, s, H-11) was related to *δ*_C_ 53.3 (C-10), *δ*_H_ 4.32 (2H, s, H-10) was associated with *δ*_C_ 53.2 (C-11)], two methylene hydrogen signals [*δ*_H_ 2.55 (2H, t, *J* = 7.4 Hz, H-8) was correlated with *δ*_C_ 31.2 (C-8), *δ*_H_ 2.84 (2H, t, *J* = 7.4 Hz, H-7) was associated with *δ*_C_ 25.7 (C-7)].

The ^13^C NMR spectrum of 2, measured in DMSO-*d*_6_ (Table 2), describes the various types of carbon signals (displayed one carboxylic acid carbonyl carbon signal at *δ*_C_ 173.1 (C-9), five sp^2^ hybrid carbon signals at *δ*_C_ 168.9 (C-4), 163.9 (C-2), 162.0 (C-6), 113.9 (C-5), 109.8 (C-3), one methoxyl carbon signal at *δ*_C_ 62.0 (C-12), and two oxymethylene carbon signals at *δ*_C_ 53.3 (C-10), 53.2 (C-11), along with two fatty carbon signals at *δ*_C_ 31.2 (C-8), 25.7 (C-7). A comparison of the 1D NMR data of 2 with Necpyrone A (Li. et al., 2015) revealed that they shared the same carbon skeleton, and the only obvious difference between them was the side chain. As a consequence, the parent nucleus of compound 2 was determined to be *α*-pyranone structure.

The HMBC spectrum (Figure 2) showed correlations of (H-8 with C-6/C-7/C-9 and H-7 with C-6/C-8/C-9), which suggested the presence of C-6 and C-7 linking to form one side chain. The HMBC cross-peaks from H-11 to C-4/C-5/C-6 indicated the presence of C-5 and C-11 linking to form another side chain. In addition, the NOESY spectrum observed that *δ*_H_ 4.26 (2H, s, H-11) was correlated with 2.84 (2H, t, *J* = 7.4 Hz, H-7), and *δ*_H_ 4.32 (2H, s, H-10) was associated with 4.06 (3H, s, 4-OCH_3_). Therefore, the structure Phomapyrone A was determined, systemically named as *3-[3,5-bis(hydroxymethyl)-4-methoxy-2-oxo-2H-Pyran-6-yl]propanoic acid*.

Compound 3 was obtained as a yellow oil (MeOH). And it had a molecular formula of C_15_H_22_O_7_ that was determined by the HRESIMS (*m/z* 314.1269 [M + Na]^+^) and NMR data, requiring 5 degrees of unsaturation. Comprehensive analysis of the ^1^H and ^13^C data of compounds 2 and 3, suggested that the basic data of its parent nucleus were basically the same, only the side chain was different. The ^1^H NMR (400 MHz, DMSO-*d*_6_)spectrum with the aid of the HSQC spectrum showed signals of hydrogen including two active hydroxyl signals *δ*_H_ 4.96 (2H, m), one methoxyl hydrogen signal [*δ*_H_ 4.06 (3H, s, 17-OCH_3_) was correlated with *δ*_C_ 62.0], three oxymethylene hydrogen signals [*δ*_H_ 4.25 (2H, d, *J* = 4.0 Hz, H-16) was related to *δ*_C_ 53.3, *δ*_H_ 4.32 (2H, d, *J* = 4.0 Hz, H-15) was associated with *δ*_C_ 53.2, *δ*_H_ 4.02 (2H, t, *J* = 6.5 Hz, H-11) was associated with *δ*_C_ 63.8], four methylene hydrogen signals [*δ*_H_ 2.63 (2H, t, *J* = 7.4 Hz, H-8) was correlated with *δ*_C_ 31.1, *δ*_H_ 2.88 (2H, t, *J* = 7.4 Hz, H-7) was associated with *δ*_C_ 25.6, *δ*_H_ 1.53 (2H, m, H-12) was associated with *δ*_C_ 30.1, *δ*_H_ 1.31 (2H, m, H-13) was associated with *δ*_C_ 18.6], one methyl hydrogen group *δ*_H_ 0.88 (3H, t, *J* = 7.3 Hz, H-14).

The ^13^C NMR spectrum (Table 2) of compound 3 demonstrated 15 carbon resonances of various types of carbon signals, displayed one carboxylic acid carbonyl carbon *δ*_C_ 171.6 (C-9), five sp^2^ hybrid carbon signals at *δ*_C_ 168.9 (C-4), 163.8 (C-2), 161.5 (C-6), 114.1 (C-5), 109.9 (C-3), one methoxyl carbon signal at *δ*_C_ 62.0 (C-17), along with eight fatty carbon signals at *δ*_C_ 63.8 (C-11), 53.3 (C-16), 53.2 (C-15), 31.1 (C-8), 30.1 (C-12), 25.6 (C-7), 18.6 (C-13), 13.5 (C-14).

The HMBC spectrum (Figure 2) showed correlations of (H-8 with C-6/C-7/C-9 and H-7 with C-5/C-6/C-8/C-9), which suggested the presence of C-6 and C-7 linking to form one side chain. The HMBC cross-peaks from H-16 to C-2/C-4/C-5 indicated C-5 and C-16 connections, and H-15 to C-2/C-3/C-4 indicated the presence of C-3 and C-15 linking to a side chain. What’s more, the correlations of (H-11 with C-9/C-12/C-13 and H-12 with C-11/C-13/C-14) suggested C-11 and C-12 connections. In addition, the NOESY spectrum observed that H-16 was correlated with H-7, and H-15 was associated with H-17. Thus, the structure Phomapyrone B was determined, systemically named *n-butyl 3-[3,5-bis(hydroxymethyl)-4-methoxy-2-oxo-2H-pyran-6-yl]propanoate*.

### 3.2 Cytotoxicity Assay

In addition, 5-fluorouridine was used as the positive control, and the cytotoxicity test results showed that compounds 1-3 and 9 exhibited moderate inhibitory effects on HL-60, with IC_50_ values of 31.02, 34.62, 27.90, and 41.07 *μ*M, respectively (Table 3). The other compounds 4–8 and 10 showed no inhibition against HL-60 with IC_50_ values greater than 50 *μ*M. Unfortunately, the compounds had no apparent effect on PC-3 or HT-29 cells. The bioactivity of compound 3 was stronger than that of compound 2, suggesting that the substitution activity of side-chain carboxylic acid was weaker than that of the ester bond. The structure-activity relationship of compounds could be further inferred by structural modification or modification of its parent nucleus.

**TABLE 3:** *In vitro* cytostatic activities of compounds (1–10).

No.	HL-60 (IC_50_ *μ*M)	PC-3 (IC_50_ *μ*M)	HT-29 (IC_50_ *μ*M)
1	31.02	>50	>50
2	34.62	>50	>50
3	27.90	>50	>50
4	>50	>50	>50
5	>50	>50	>50
6	>50	>50	>50
7	>50	>50	>50
8	>50	>50	>50
9	41.07	>50	>50
10	>50	>50	>50
5-Fluorouridine	6.38	7.77	5.82

## 4 Conclusion

In conclusion, three new compounds, 11*S,* 13*R*-(+)-phomacumarin A (1), phomapyrone A (2), and phomapyrone B (3) were isolated from the endophytic fungus *Phoma* sp. YN02-P-3. The new compounds have a moderate inhibitory effect on human acute promyelocytic leukemia cell HL-60. Our experiment expected to obtain more novel pyranone compounds under the condition of changing the culture conditions, but after replacing rice medium with corn medium, the main secondary metabolites of YN02-P-3 changed from pyranone compounds to aromatic compounds. Although the desired products were not obtained, it provided a new idea for stimulating fungi to get more novel compounds by changing the fermentation conditions in the future and making full use of microbial resources.

In recent years, more and more attention has been paid to sustainable development (Rigobelo et al., 2021; Garuso et al., 2020; Berestetskiy et al., 2020). People have begun to interpret ecology and explore the potential of endophytic interactions in plant growth, and pay more and more attention to the ecological role of endophytic fungal secondary metabolites (Carvalho et al., 2020; Amirzakariya and Shakeri, 2022). In order to obtain secondary metabolites sustainably, protect the ecological environment and guard human health (Khanam et al., 2020; Bhadra et al., 2022). Therefore, to search for bioactive secondary metabolites from endophytic fungi and further explore their medicinal value is not only the work of researchers but also of great significance to protect human health and the ecological environment.

## Data Availability

The datasets presented in this study can be found in online repositories. The names of the repository/repositories and accession number(s) can be found in the article/Supplementary Material.
