# Supplementary material for: Pyranone Derivatives With Antitumor Activities, From the Endophytic Fungus Phoma sp. YN02-P-3
Source: Front Chem. 2022 Jul 7;10:950726. doi: 10.3389/fchem.2022.950726 (PMC9300907; doi:10.3389/fchem.2022.950726)
Supplement: Supplementary file 1 [file DataSheet1.pdf]

Supplementary Material

The strain YN02-P-3 was identified as *Phoma* sp. by MEGE 4 software and compared with GENBANK on NCBI website, the homology of *medicaginis* was 100%. The ITS sequence of the strain was as follows:

TGCGGAAGGATCATTACCTAGAGTTGTAGGCTTTGCCTGCTATCTCTTACCCATGTCTTTT
AAGTACCTTACGTTTCCTCGGCGGGTCCGCCCGCCGATTGGACAATTTAAACCATTTGCA
GTTGCAATCAGCGTCTGAAAAAACTTAATAGTTACAACTTTCAACAACGGATCTCTTGGT
TCTGGCATCGATGAAGAACGCAGCGAAATGCGATAAGTAGTGTGAATTGCAGAATTCAG
TGAATCATCGAATCTTTGAACGCACATTGCGCCCCTTGGTATTCCATGGGGCATGCCTGT
TCGAGCGTCATTTGTACCTTCAAGCTCTGCTTGGTGTTGGGTGTTTGTCTCGCCTCTGCGT
GTAGACTCGCCTCAAAACAATTGGCAGCCGGCGTATTGATTTCGGAGCGCAGTACATCT
CGCGCTTTGCACTCATAACGACGACGTCCAAAAGTACATTTTTACACTCTTGACCTCGGA
TCAGGTAGGGATACCCGCTGAACTTAAGCATATCAAT

Meas. m/z	#	Formula	m/z	err [ppm]	Mean err [ppm]	rdb	N-Rule	e¯ Conf	mSigma	Std I	Std Mean m/z	Std I VarNorm	Std m/z Diff	Std Comb Dev
289.1439														
	1	C 17 H 21 O 4	289.1434	-1.6	-1.7	7.5	ok	even	22.82	0.0408	0.0006	0.0168	0.0005	0.8427

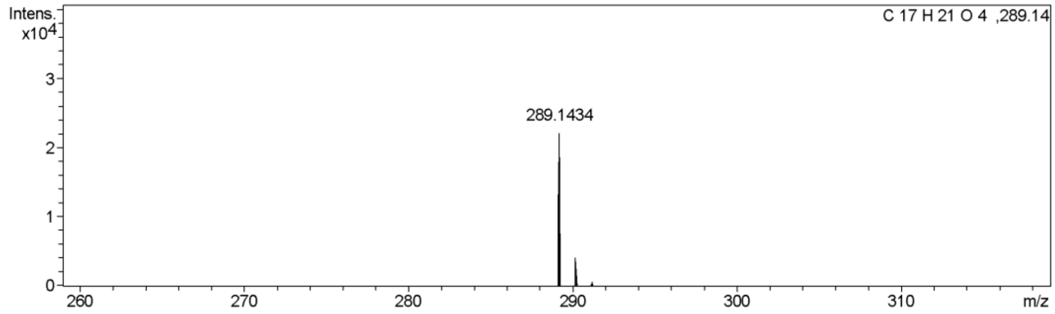

Meas. m/z	#	Formula	m/z	err [ppm]	Mean err [ppm]	rdb	N-Rule	e¯ Conf	mSigma	Std I	Std Mean m/z	Std I VarNorm	Std m/z Diff	Std Comb Dev

Figure S1. HR-ESI-MS spectrum of compound **1**.

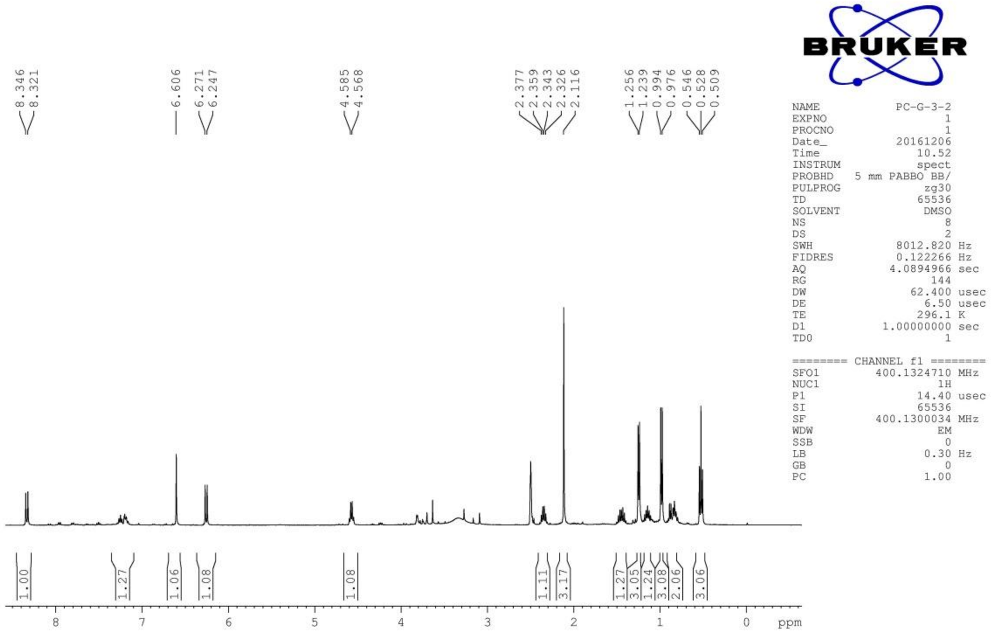

Figure S2. ^{1}H NMR (400 MHz, DMSO-d_6) spectrum of compound **1**.

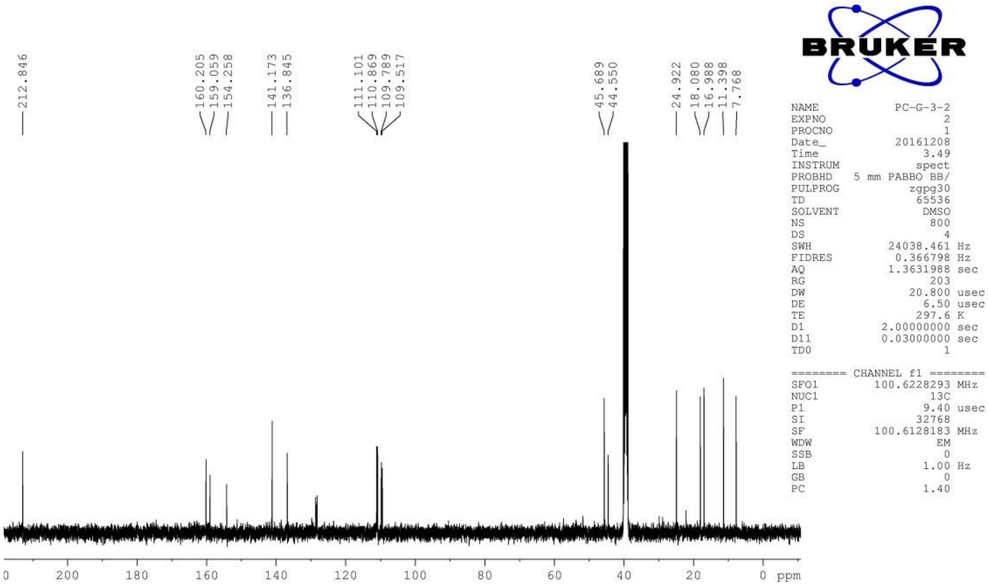

Figure S3. ^{13}C NMR (100 MHz, DMSO-d_6) spectrum of compound **1**.

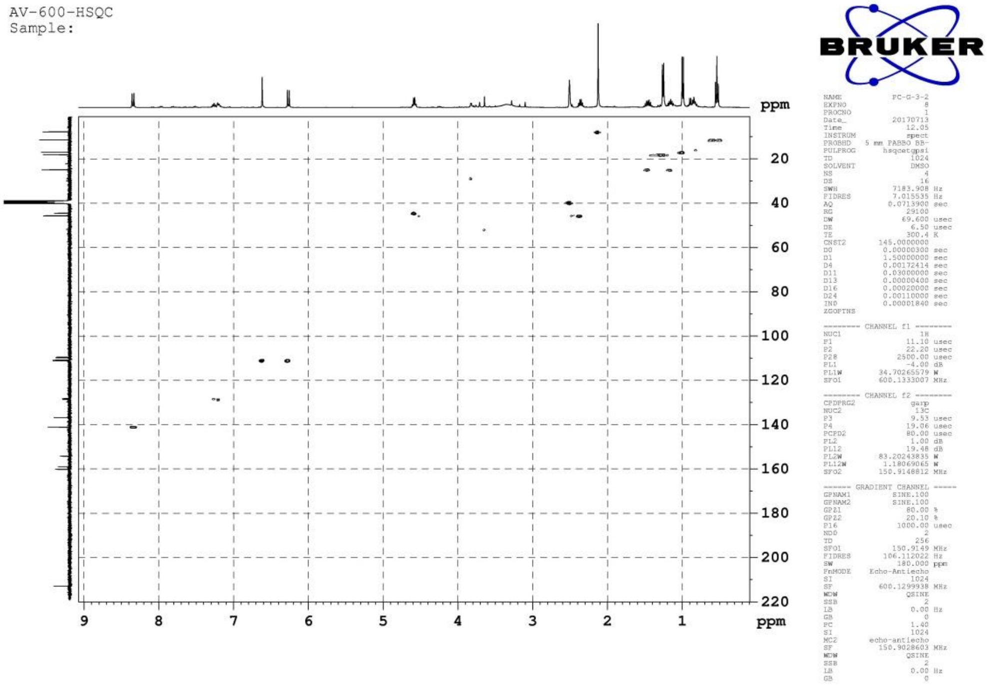

Figure S4. HSQC (600 MHz, DMSO-d_6) spectrum of compound **1**.

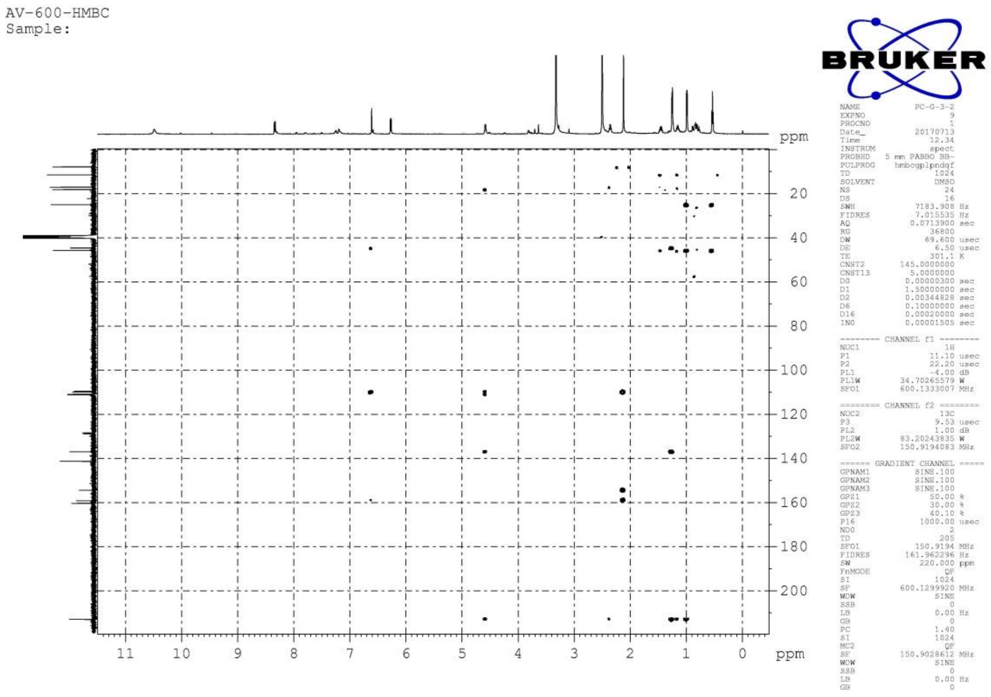

Figure S5. HMBC (600 MHz, DMSO-d_6) spectrum of compound **1**.

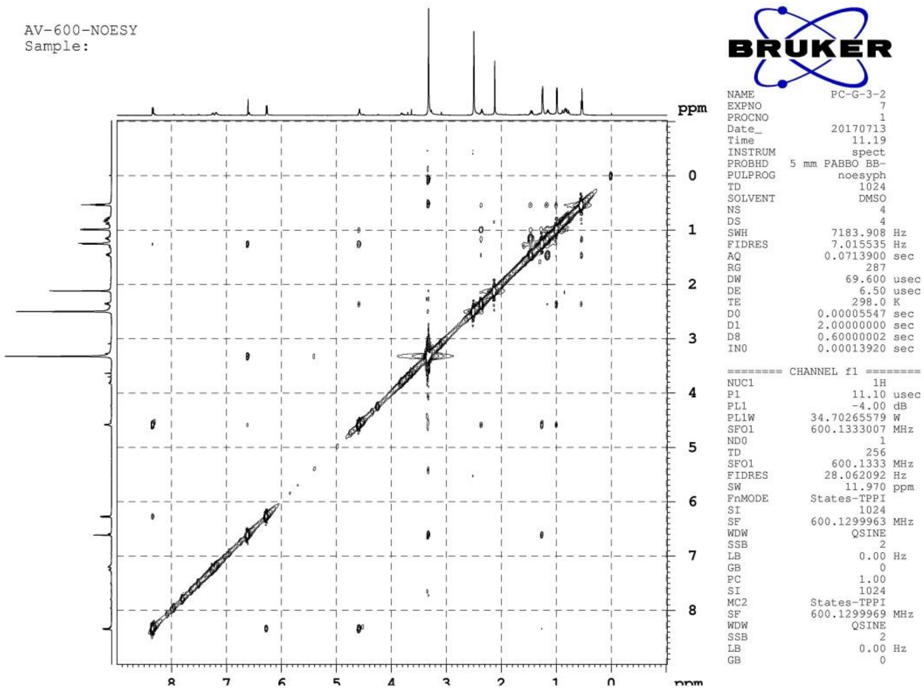

Figure S6. NOESY (600 MHz, DMSO-d_6) spectrum of compound **1**.

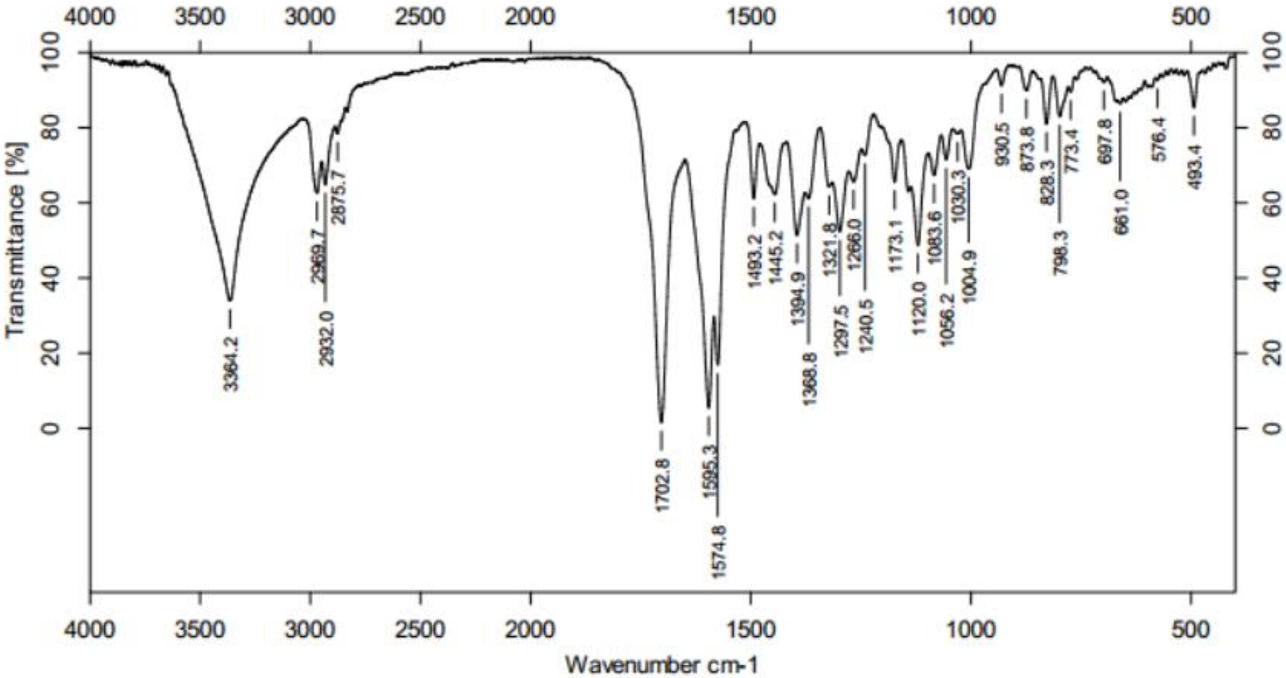

Figure S7. IR spectrum of compound **1**.

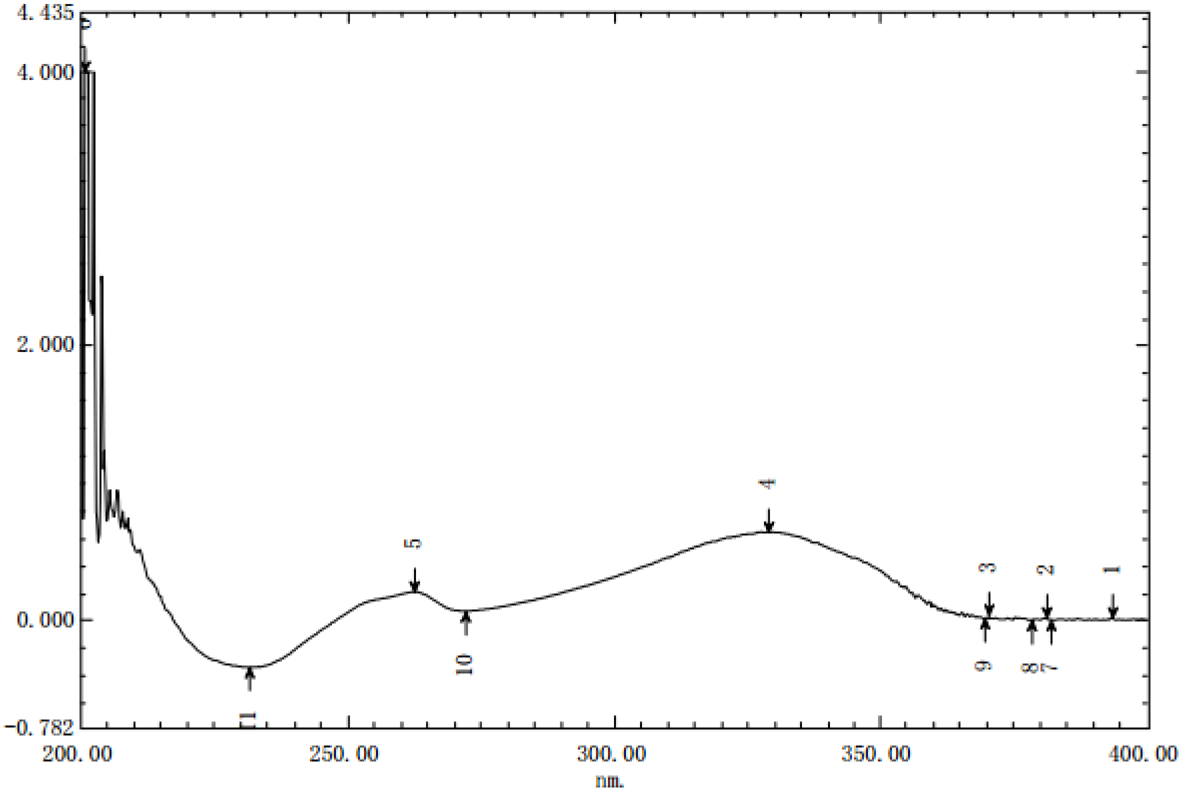

Figure S8. UV spectrum of compound **1**.

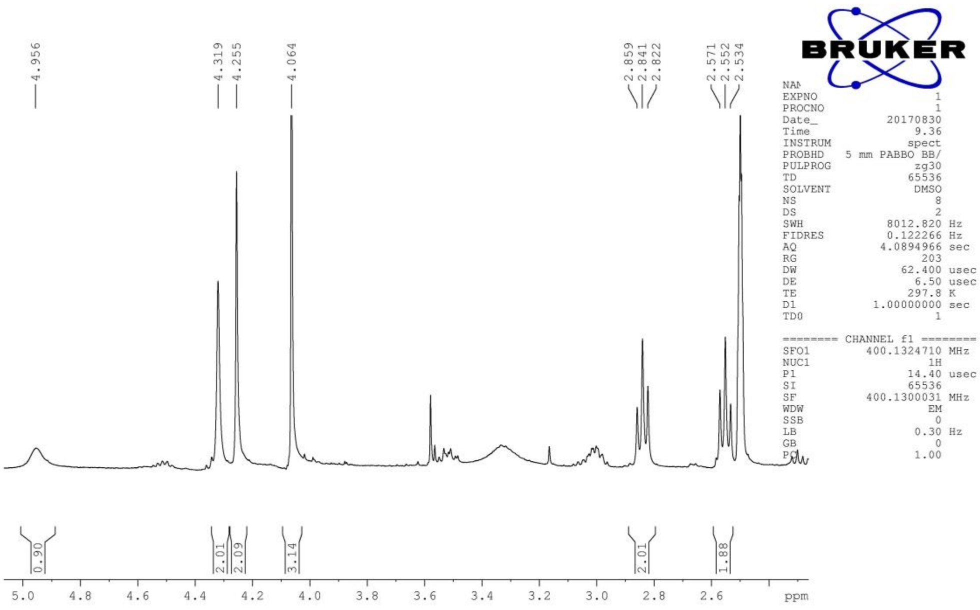

Figure S9. ^{1}H NMR (400 MHz, DMSO-d_6) spectrum of compound **2**.

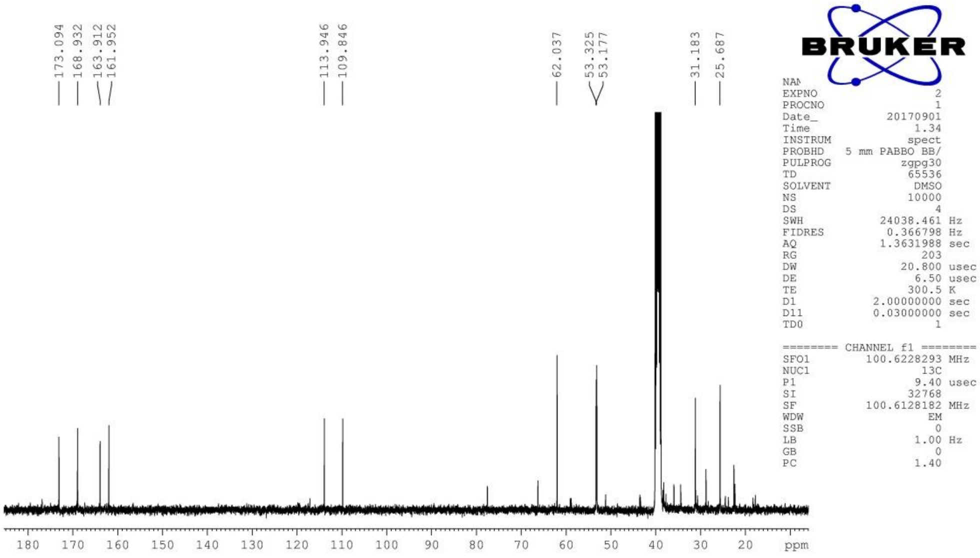

Figure S10. ^{13}C NMR (100 MHz, DMSO-d_6) spectrum of compound **2**.

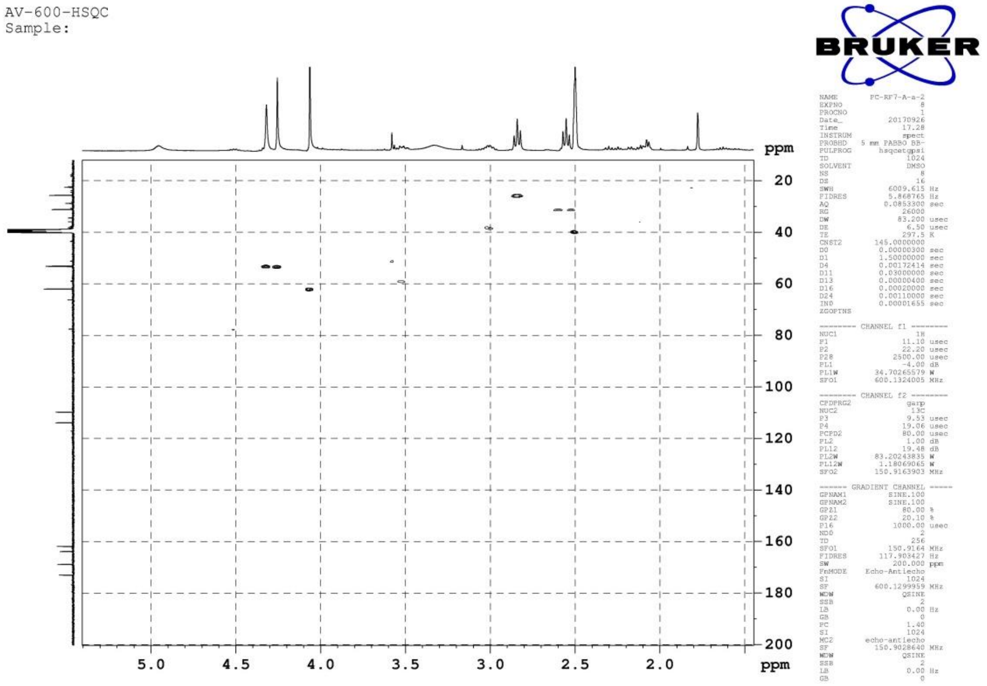

Figure S11. HSQC (600 MHz, DMSO-d_6) spectrum of compound **2**.

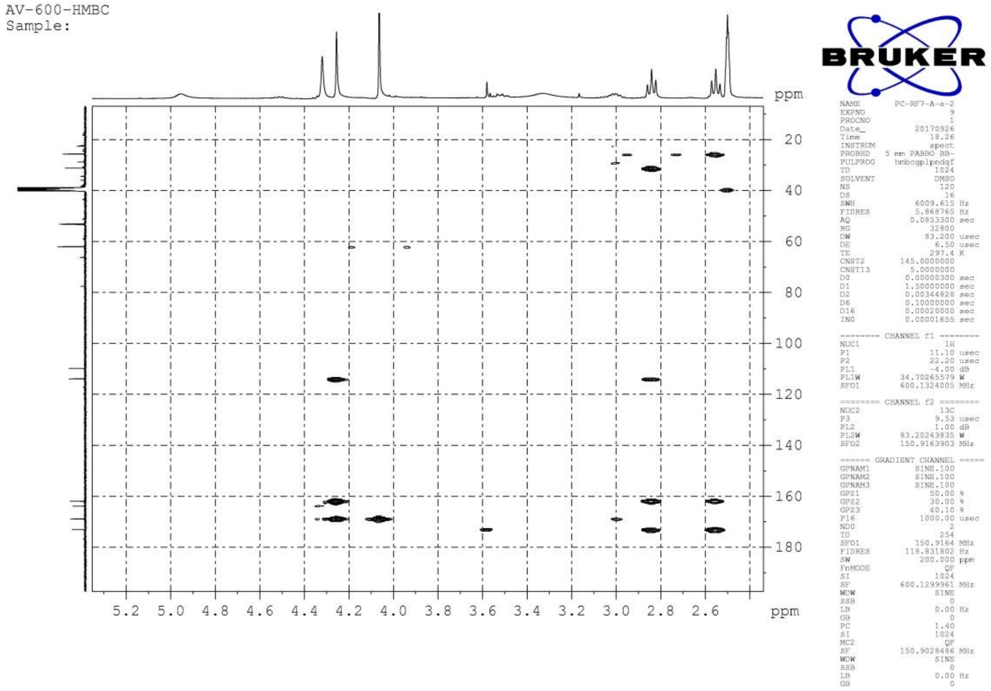

Figure S12. HMBC (600 MHz, DMSO-d_6) spectrum of compound **2**.

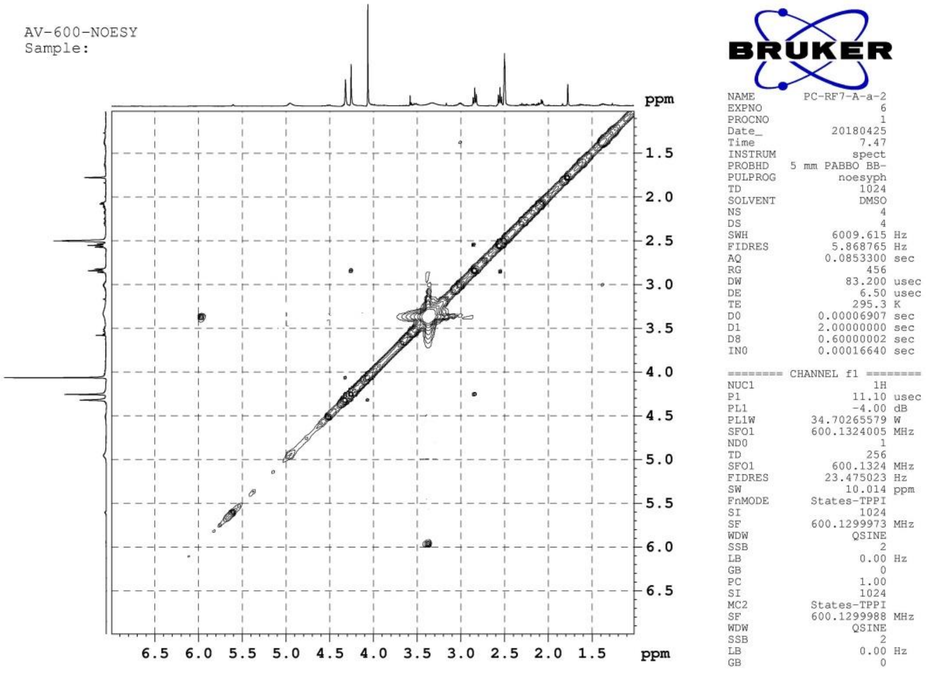

Figure S13. NOESY (600 MHz, DMSO-d_6) spectrum of compound **2**.

Meas. m/z	#	Formula	m/z	err [ppm]	Mean err [ppm]	rdb	N-Rule	e⁻ Conf	mSigma	Std I	Std Mean m/z	Std I VarNorm	Std m/z Diff	Std Comb Dev
281.0665														
	1	C 11 H 14 Na O 7	281.0632	-11.7	-12.1	4.5	ok	even	14.17	0.0316	0.0034	0.0164	0.0015	0.8427
285.1893														
	1	C 13 H 25 N 4 O 3	285.1921	10.0	-0.8	3.5	ok	even	66.45	0.1271	0.0090	0.0698	0.0015	0.8427

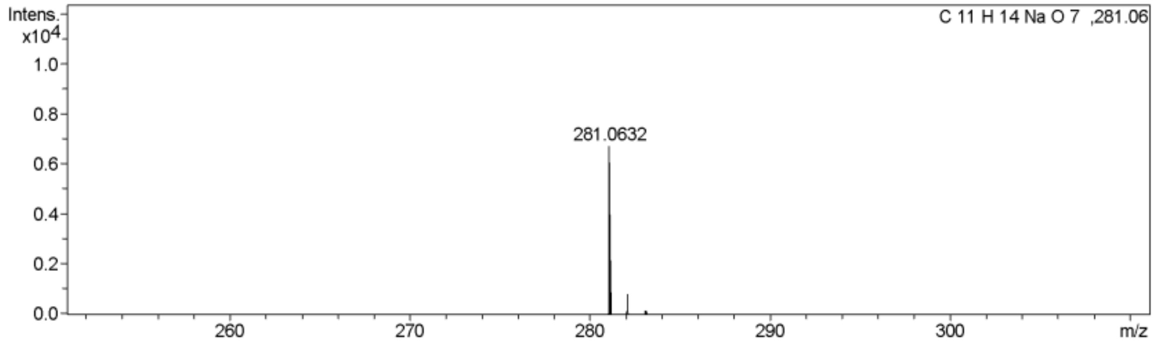

Figure S14. HR-ESI-MS spectrum of compound **2**.

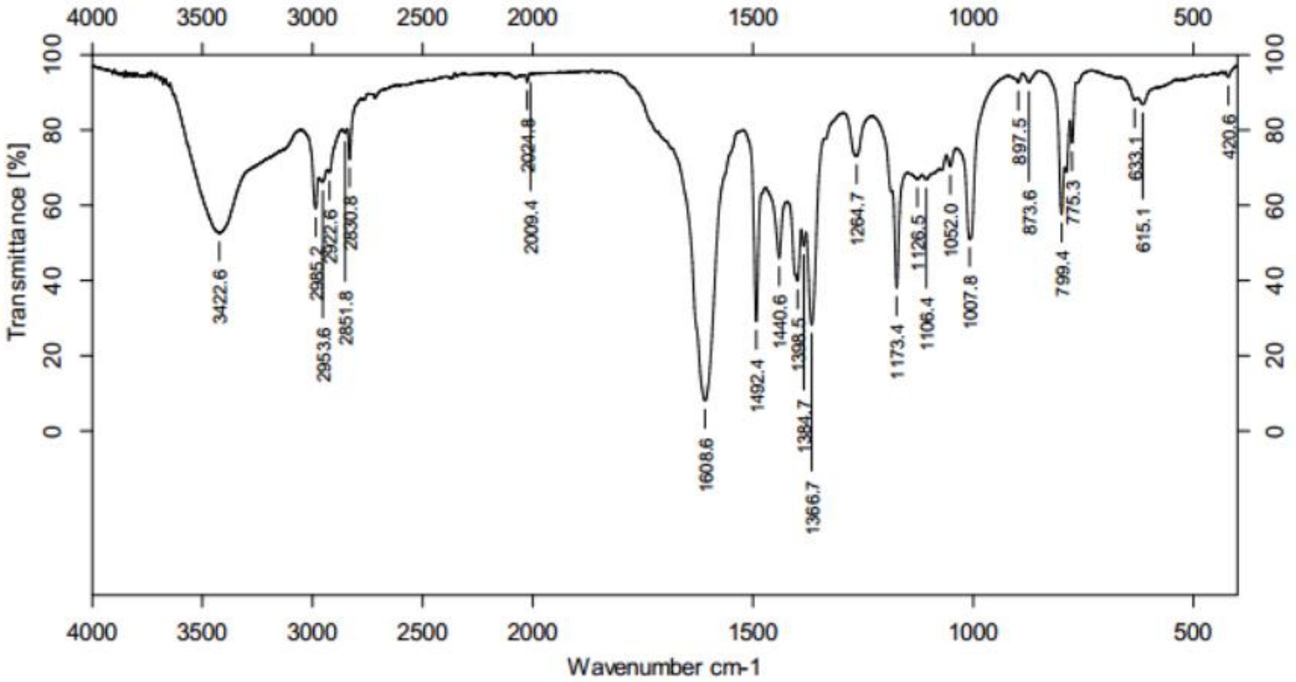

Figure S15. IR spectrum of compound **2**.

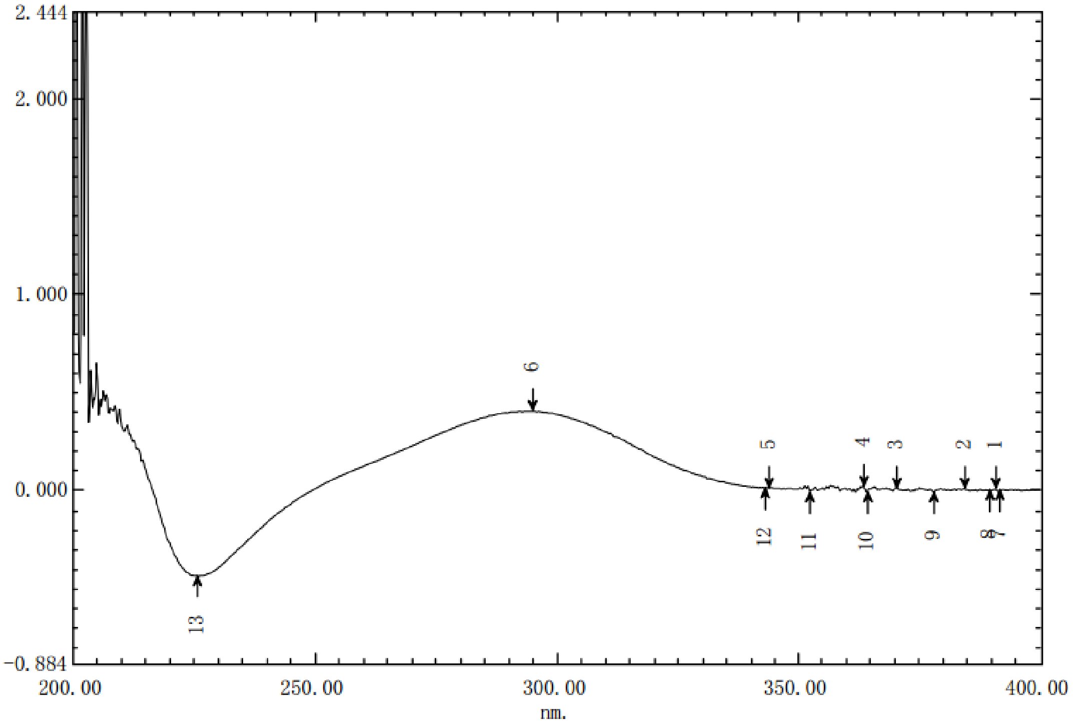

Figure S16. UV spectrum of compound **2**.

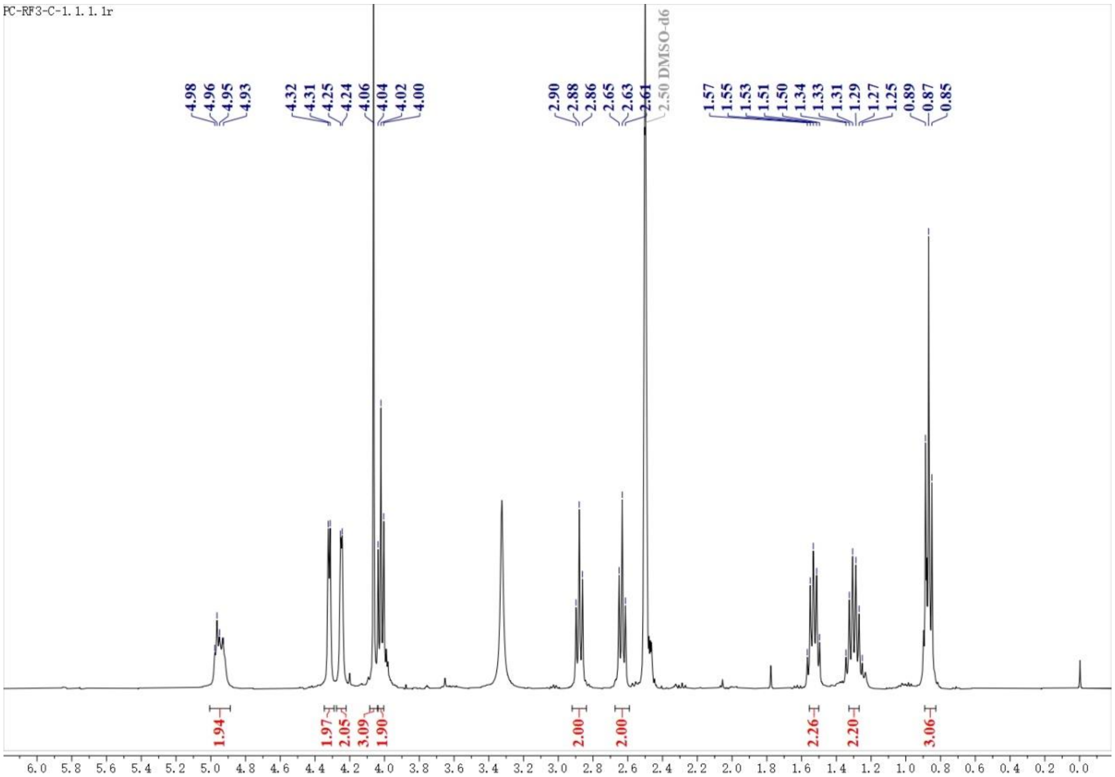

Figure S17. ^{1}H NMR (400 MHz, DMSO-d_6) spectrum of compound **3**.

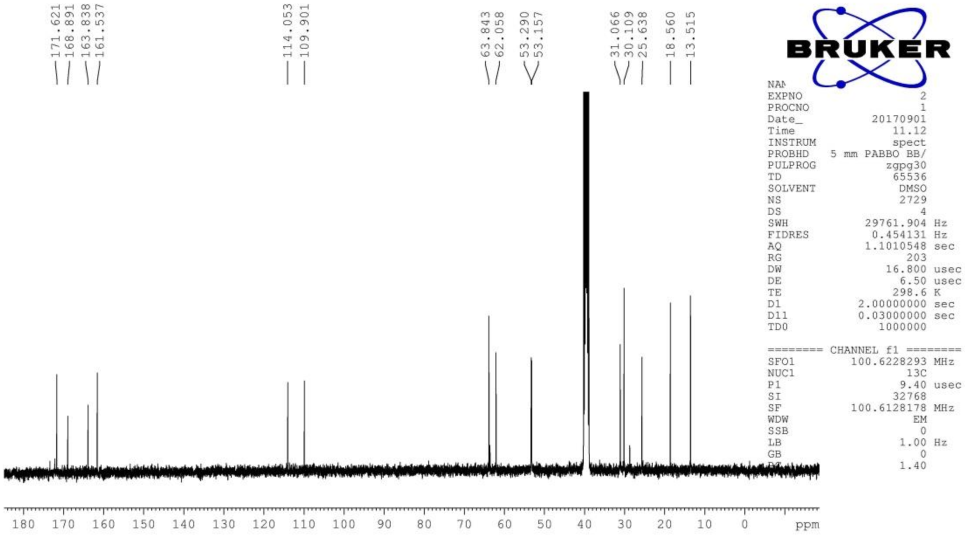

Figure S18. ^{13}C NMR (100 MHz, DMSO-d_6) spectrum of compound **3**.

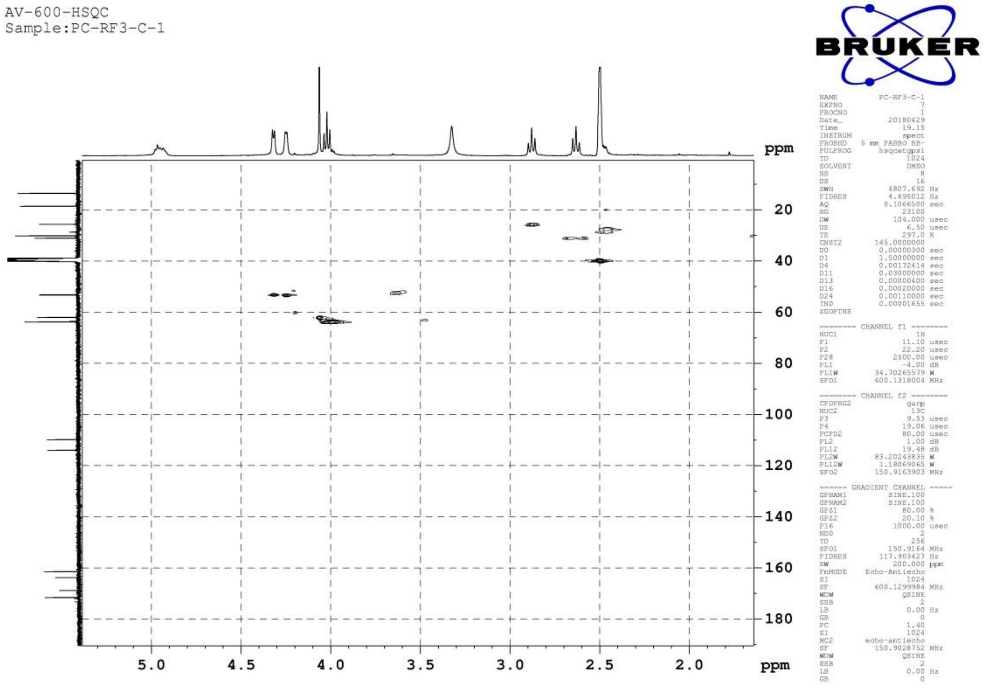

Figure S19. HSQC (600 MHz, DMSO-d_6) spectrum of compound **3**.

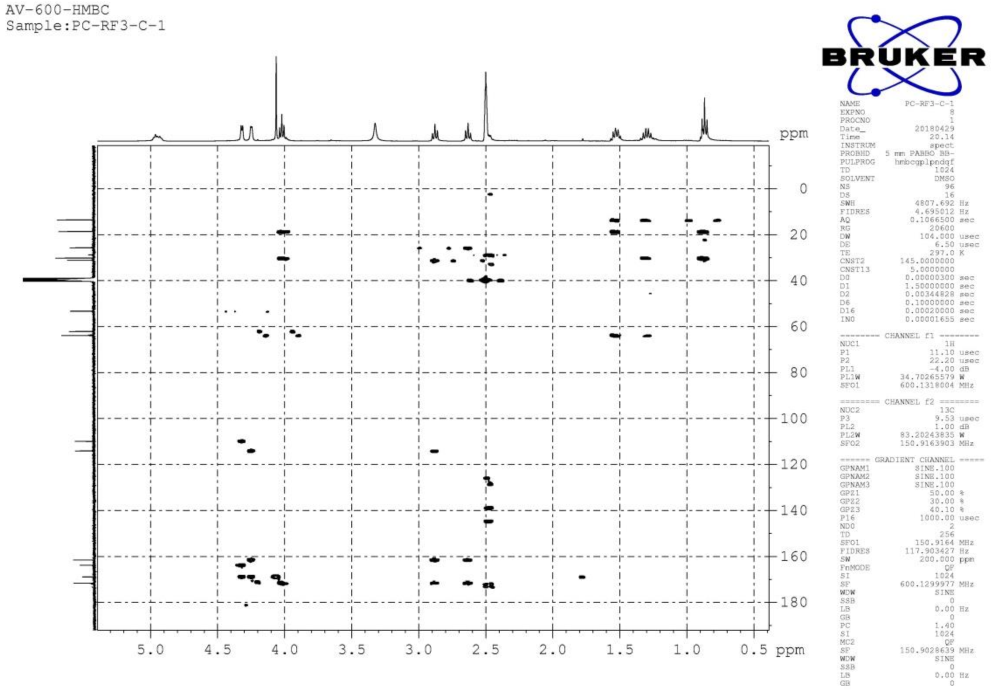

Figure S20. HMBC (600 MHz, DMSO-d_6) spectrum of compound **3**.

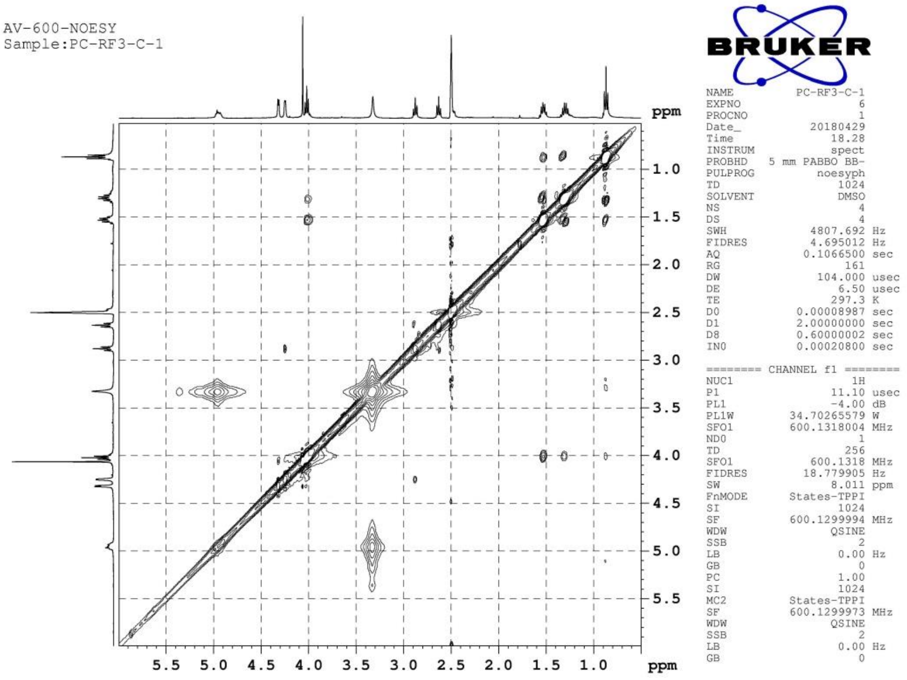

Figure S21. NOESY (600 MHz, DMSO-d_6) spectrum of compound **3**.

Meas. m/z	#	Formula	m/z	err [ppm]	Mean err [ppm]	rdb	N-Rule	e¯ Conf	mSigma	Std I	Std Mean m/z	Std I VarNorm	Std m/z Diff	Std Comb Dev
337.1269														
	1	C 15 H 22 Na O 7	337.1258	-3.4	-3.8	4.5	ok	even	18.48	0.0326	0.0015	0.0216	0.0002	0.8427

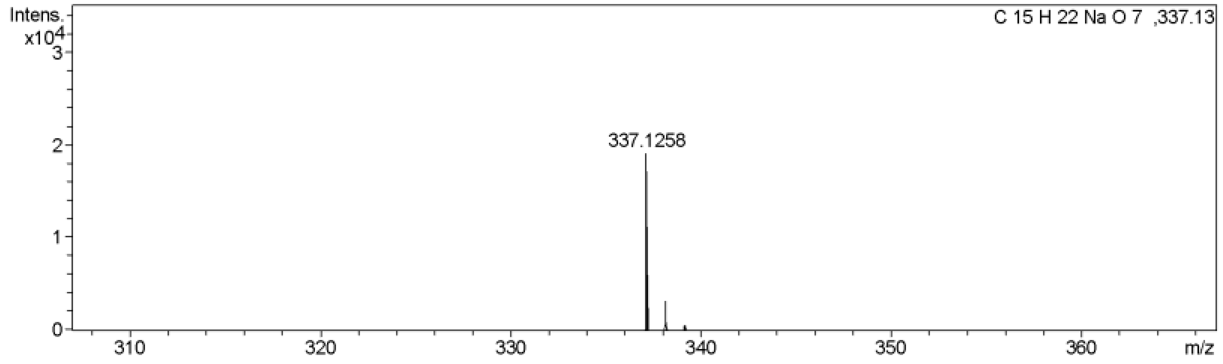

Figure S22. HR-ESI-MS spectrum of compound **3**.

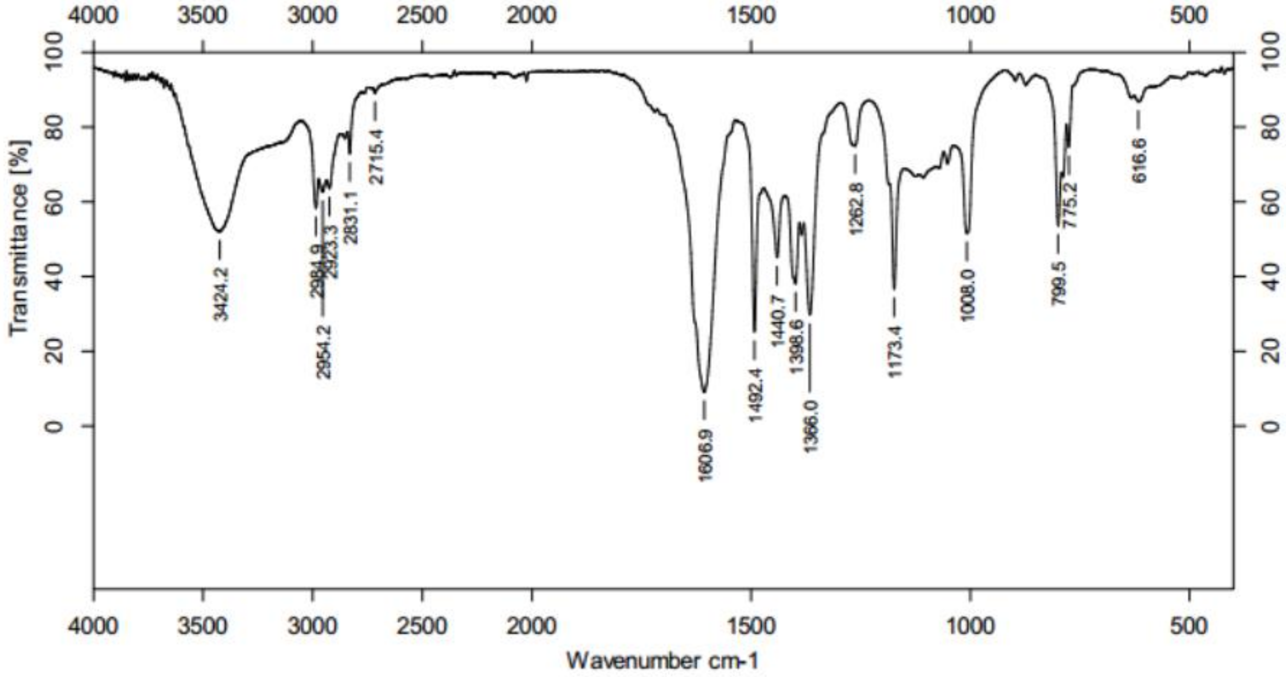

Figure S23. UV spectrum of compound **3**.

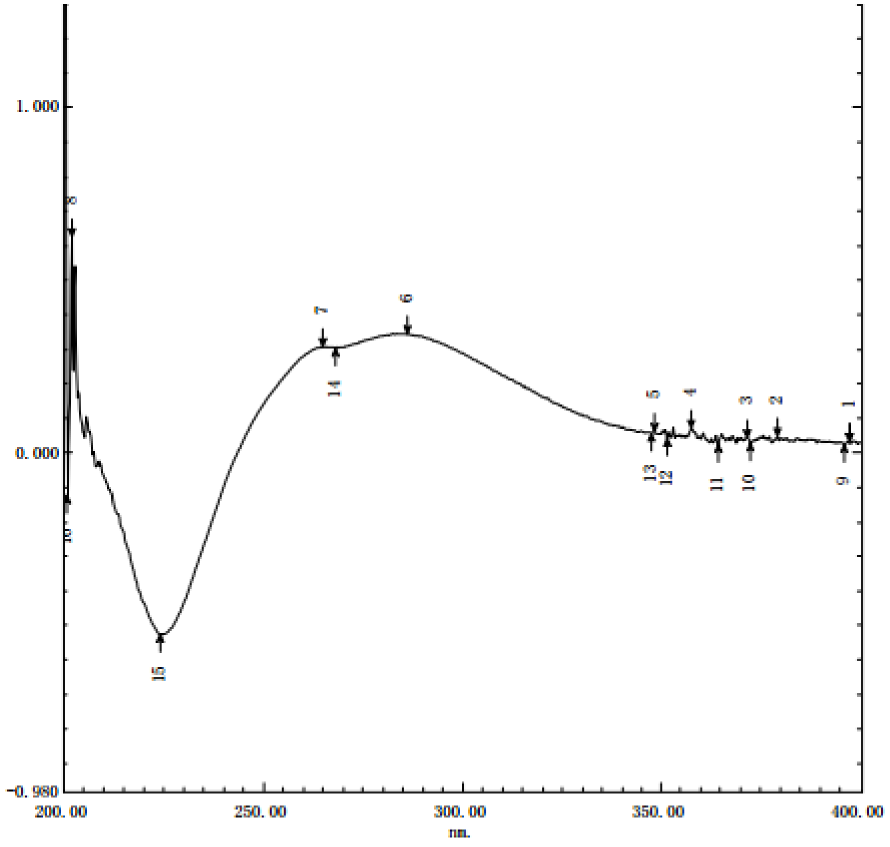

Figure S24. UV spectrum of compound **3**.

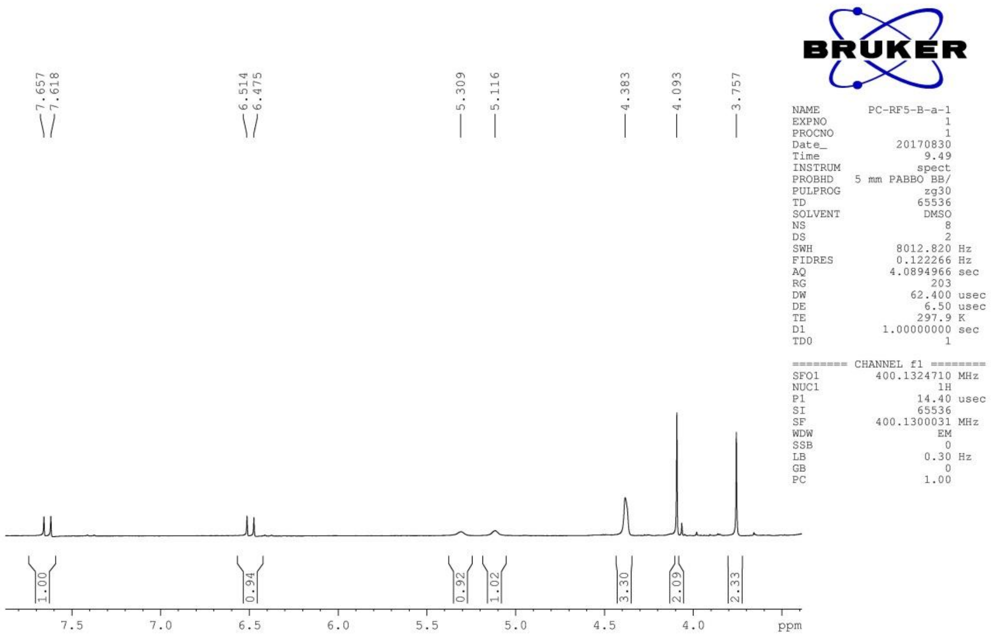

Figure S25. ^{1}H NMR (400 MHz, DMSO-d_6) spectrum of compound **4**.

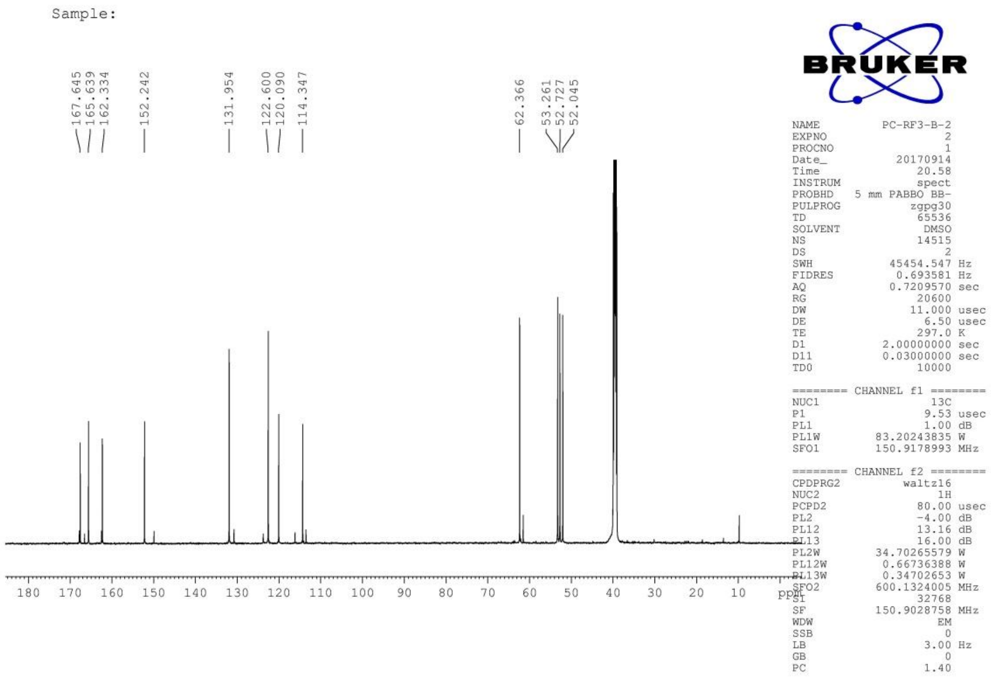

Figure S26. ^{13}C NMR (100 MHz, DMSO-d_6) spectrum of compound **4**.

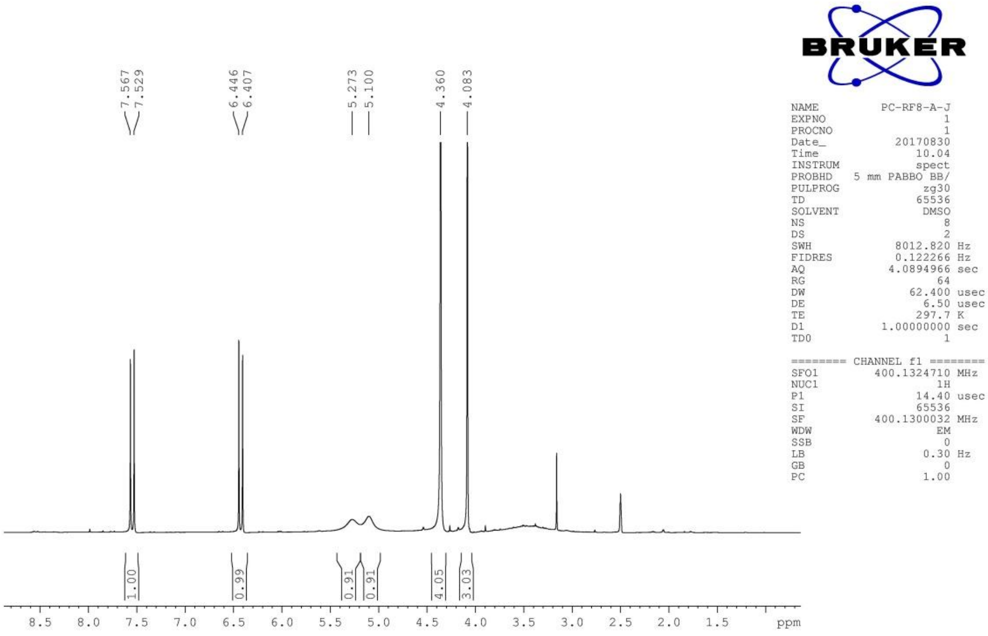

Figure S27. ^{1}H NMR (400 MHz, DMSO-d_6) spectrum of compound **5**.

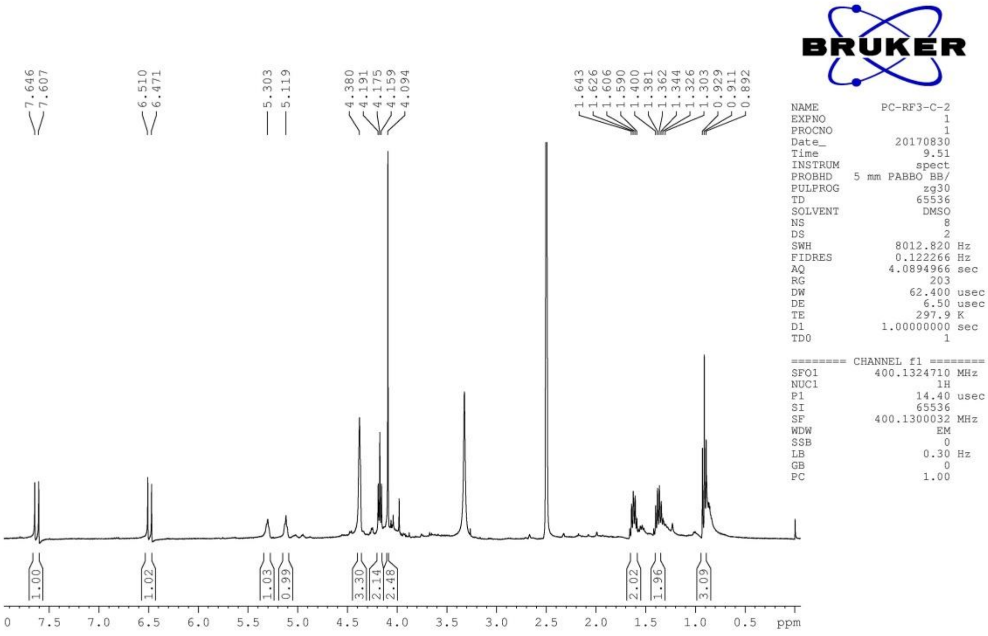

Figure S28. ^{1}H NMR (400 MHz, DMSO-d_6) spectrum of compound **6**.

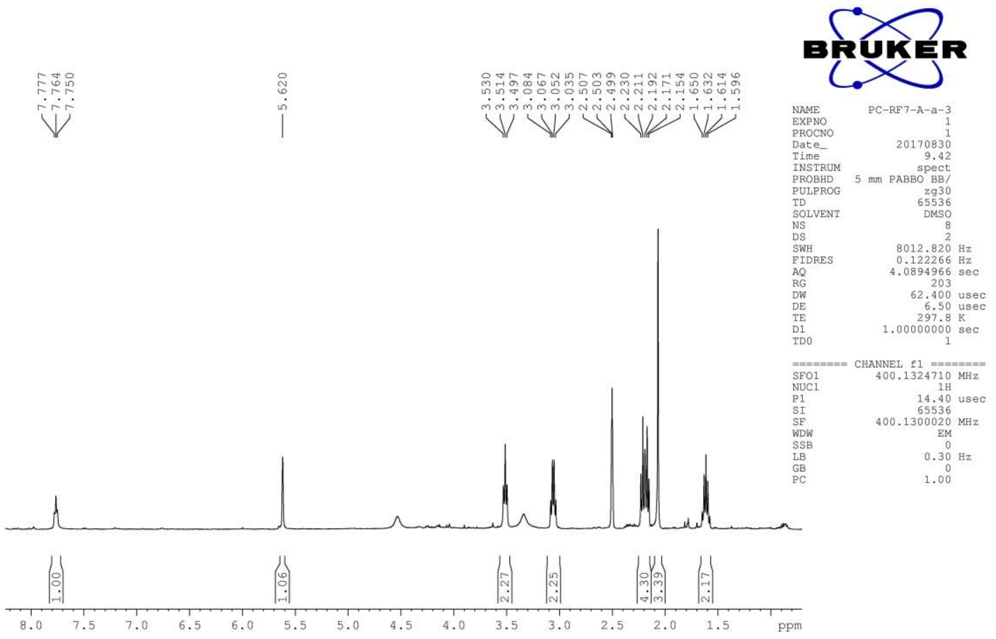

Figure S29. ^{1}H NMR (400 MHz, DMSO-d_6) spectrum of compound **7**.

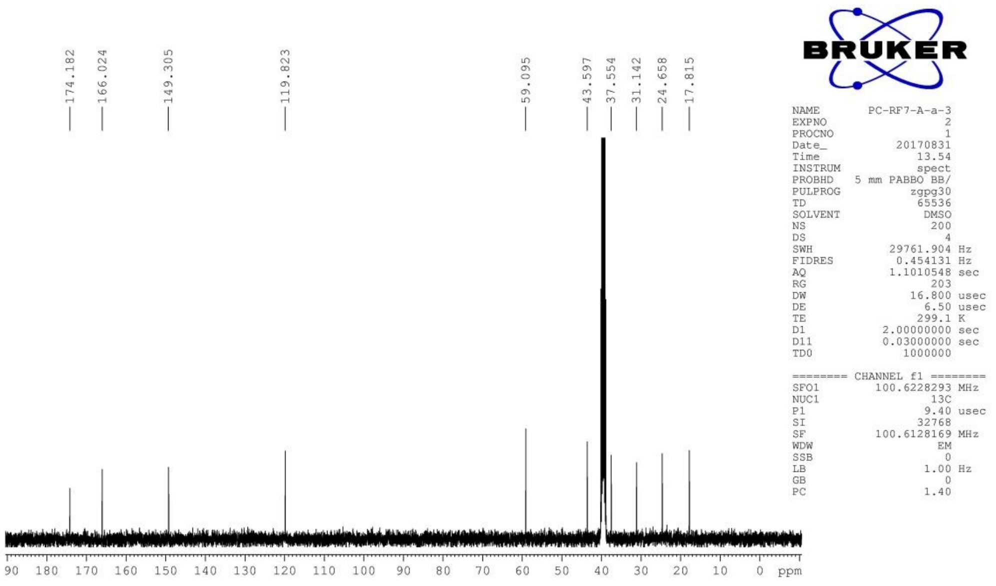

Figure S30. ^{13}C NMR (100 MHz, DMSO-d_6) spectrum of compound **7**.

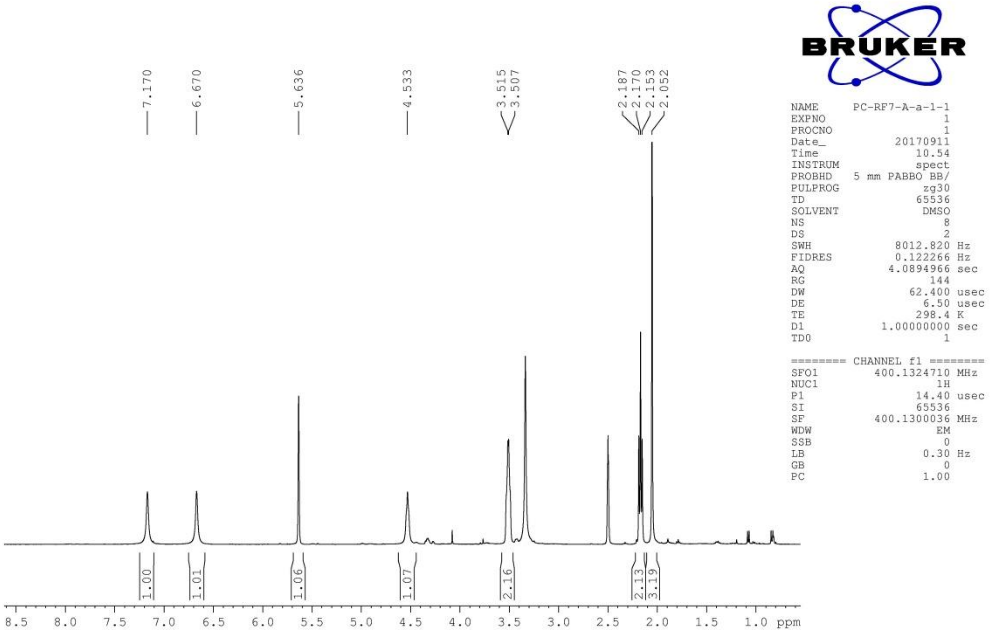

Figure S31. ^{1}H NMR (400 MHz, DMSO-d_6) spectrum of compound **8**.

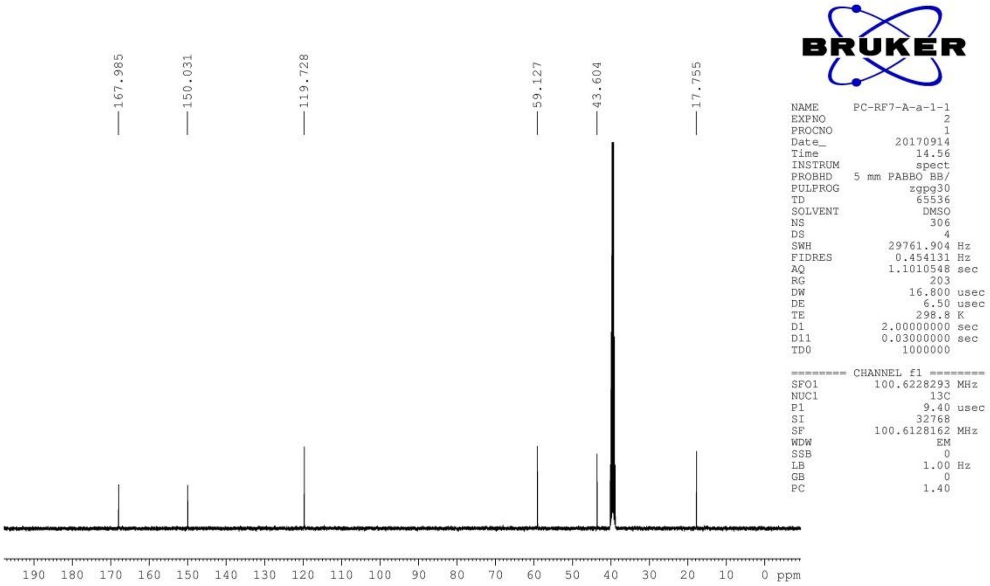

Figure S32. ^{13}C NMR (100 MHz, DMSO-d_6) spectrum of compound **8**.

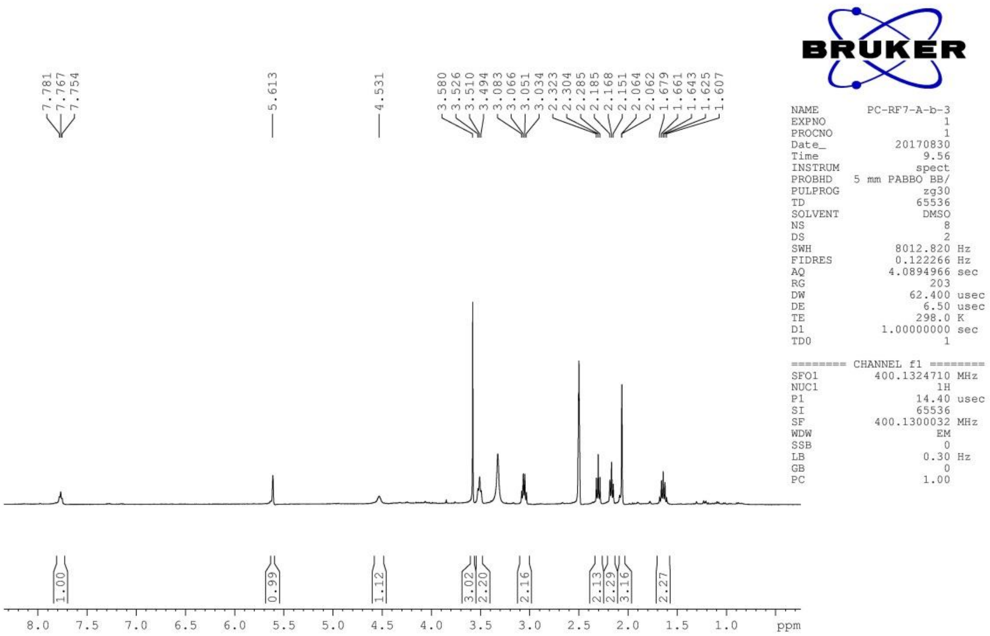

Figure S33. ^{1}H NMR (400 MHz, DMSO-d_6) spectrum of compound **9**.

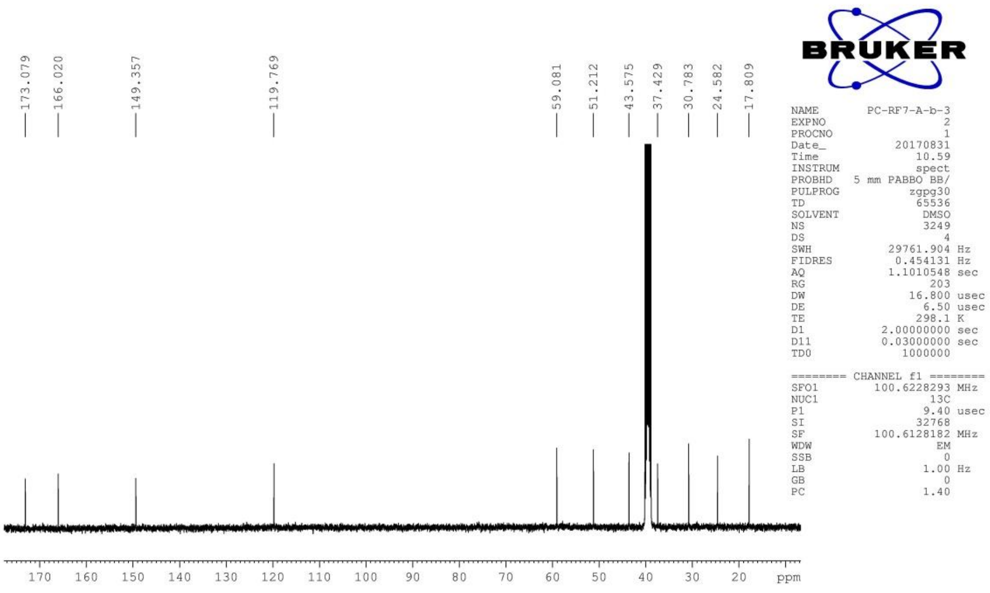

Figure S34. ^{13}C NMR (100 MHz, DMSO-d_6) spectrum of compound **9**.

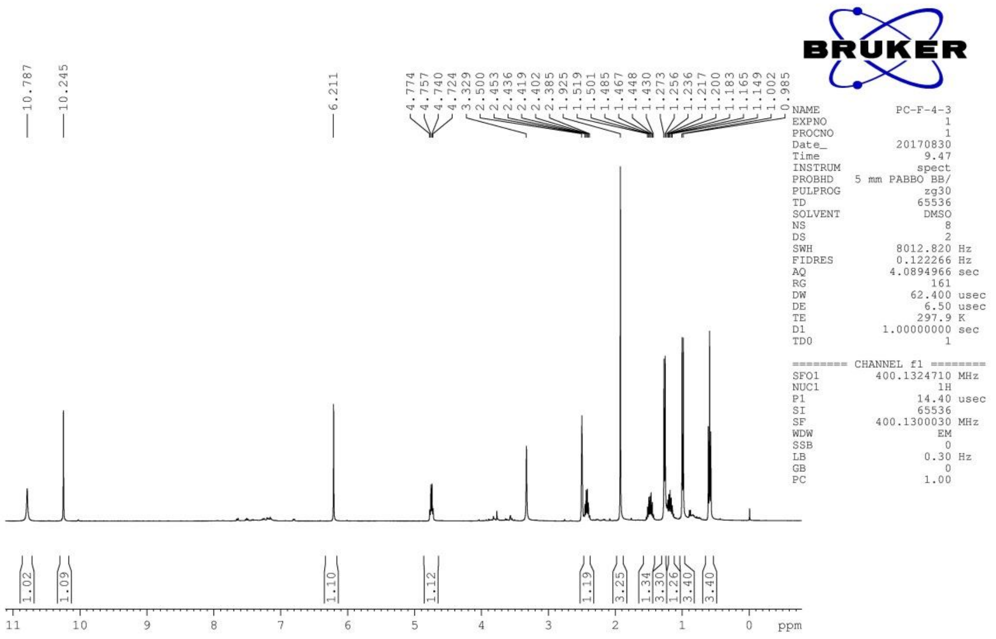

Figure S35. ^{1}H NMR (400 MHz, DMSO-d_6) spectrum of compound **10**.

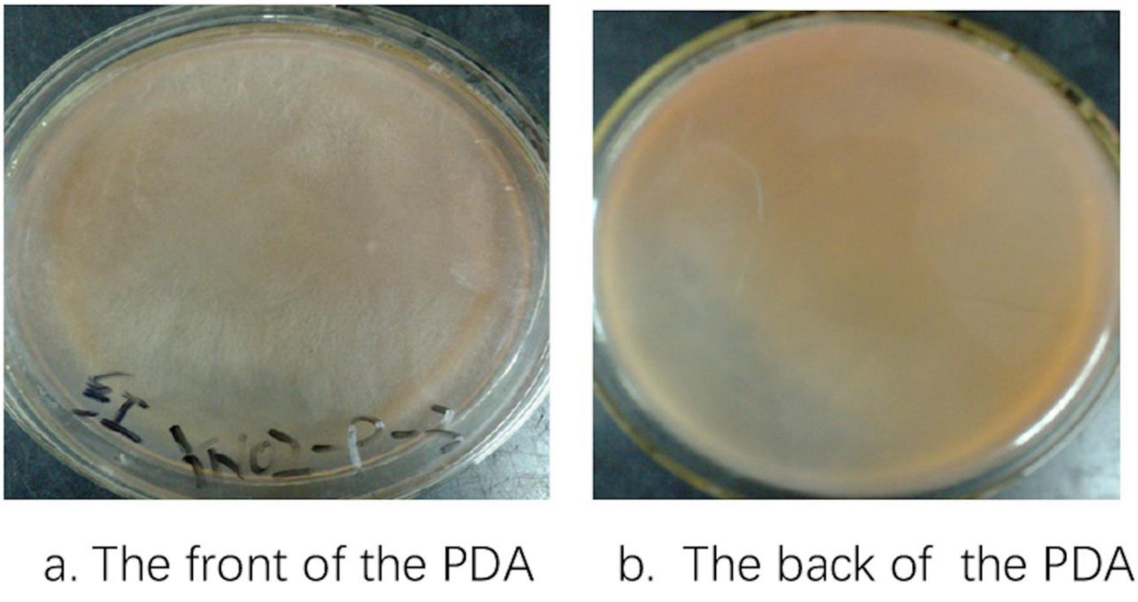

Figure S36. The morphological diagram of YN02-P-3.

9*S*, 11*R*-(+)-ascosalitoxin (11)

Yellow oil (MeOH); $[\alpha]_D^{20}$ +149.5 (c 0.1, MeOH).
